# JNK signaling prevents biliary cyst formation through a CASPASE-8–dependent function of RIPK1 during aging

**DOI:** 10.1073/pnas.2007194118

**Published:** 2021-03-08

**Authors:** Katrin Müller, Hanna Honcharova-Biletska, Christiane Koppe, Michèle Egger, Lap Kwan Chan, Anne T. Schneider, Lena Küsgens, Friederike Böhm, Yannick Boege, Marc E. Healy, Johannes Schmitt, Sarah Comtesse, Mirco Castoldi, Christian Preisinger, Marta Szydlowska, Enrico Focaccia, Nadine T. Gaisa, Sven H. Loosen, Simone Jörs, Frank Tacke, Christoph Roderburg, Verena Keitel, Johannes G. Bode, Peter Boor, Roger J. Davis, Thomas Longerich, Fabian Geisler, Mathias Heikenwalder, Achim Weber, Mihael Vucur, Tom Luedde

**Affiliations:** ^a^Division of Biliary and Gastrointestinal Oncology, University Hospital Rheinisch-Westfälische Technische Hochschule (RWTH) Aachen, 52074 Aachen, Germany;; ^b^Department of Pathology and Molecular Pathology, University of Zurich and University Hospital Zurich, 8091 Zurich, Switzerland;; ^c^Department of Gastroenterology, Hepatology and Infectious Diseases, University Hospital Duesseldorf, Medical Faculty, Heinrich Heine University, 40225 Duesseldorf, Germany;; ^d^Proteomics Core Facility, Interdisciplinary Center for Clinical Research, University Hospital RWTH Aachen, 52074 Aachen, Germany;; ^e^Division of Chronic Inflammation and Cancer, German Cancer Research Center (Deutsches Krebsforschungszentrum), 69120 Heidelberg, Germany;; ^f^Institute of Pathology, University Hospital RWTH Aachen, 52074 Aachen, Germany;; ^g^Internal Medicine II, Klinikum rechts der Isar, Technical University Munich, 81675 Munich, Germany;; ^h^Department of Hepatology and Gastroenterology, Charité University Medical Center Berlin, 10117 Berlin, Germany;; ^i^Department of Nephrology and Immunology, University Hospital RWTH Aachen, 52074 Aachen, Germany;; ^j^Program in Molecular Medicine, University of Massachusetts Medical School, Worcester, MA 01605;; ^k^Institute of Pathology, University Hospital Heidelberg, 69120 Heidelberg, Germany;; ^l^Institute of Molecular Cancer Research, University of Zurich, 8057 Zurich, Switzerland

**Keywords:** cholangiocytes, liver cysts, liver, programmed cell death, MK2

## Abstract

The c-Jun N-terminal kinase (JNK) signaling pathway mediates adaptation to stress signals and has been associated with cell death, cell proliferation, and malignant transformation in the liver. However, up to now, its function was experimentally studied mainly in young mice. By generating mice with combined conditional ablation of *Jnk1* and *Jnk2* in liver parenchymal cells (LPCs) (JNK1/2^LPC-KO^ mice; KO, knockout), we unraveled a function of the JNK pathway in the regulation of liver homeostasis during aging. Aging JNK1/2^LPC-KO^ mice spontaneously developed large biliary cysts that originated from the biliary cell compartment. Mechanistically, we could show that cyst formation in livers of JNK1/2^LPC-KO^ mice was dependent on receptor-interacting protein kinase 1 (RIPK1), a known regulator of cell survival, apoptosis, and necroptosis. In line with this, we showed that RIPK1 was overexpressed in the human cyst epithelium of a subset of patients with polycystic liver disease. Collectively, these data reveal a functional interaction between JNK signaling and RIPK1 in age-related progressive cyst development. Thus, they provide a functional linkage between stress adaptation and programmed cell death (PCD) in the maintenance of liver homeostasis during aging.

## Significance

JNK signaling has been studied intensively in models of liver physiology and disease, but previous studies had focused on young mice. However, it had not been recognized that JNK plays a fundamental role in maintaining liver homeostasis and preventing the formation of biliary cysts in aging mice. These observations call for caution in all long-term pharmacological inhibition strategies targeting the JNK pathway. Finally, our results provide evidence of a molecular link between JNK and the cell-death mediator RIPK1. The specific overexpression of RIPK1 in cysts of a subset of patients with polycystic liver disease suggests that RIPK1 might be mechanistically involved in the pathogenesis of human biliary cysts.

While in the normal liver almost all cells are in a dormant G0 phase with low turnover, this condition is disrupted by chronic liver disease in which viral, toxic, metabolic, or autoimmune injury leads to hepatocellular death, followed by inflammation and compensatory hepatocyte proliferation. Compensatory proliferation of liver cells is a strictly regulated process that normally stops when an underlying pathogenic stimulus is removed and functional liver mass and architecture are fully restored. In line with this, an imbalance between cell death and proliferation can have serious effects on the liver, and an improper adjustment of compensatory proliferation in chronic hepatitis is a hallmark of liver cancer development (1).

Mitogen-activated protein kinases (MAPKs) are a family of signaling molecules that are essential for linking extracellular stress signals and cell death with proliferation. The c-Jun N-terminal kinase (JNK) family is one of the MAPKs activated by tumor necrosis factor (TNF) (2). It is encoded by three genes, *Jnk1*, *Jnk2*, and *Jnk3*; the products of two of these genes, JNK1 and JNK2, are expressed in the liver (3). Previous studies suggested that JNK signaling may play an important role in promoting the death and/or proliferation of hepatocytes, but these results were controversial in part due to different strategies to inhibit this pathway in vivo and the overall genetic context [e.g., nuclear factor κB proficiency versus deficiency (3)]. Mice with whole-body ablation of either *Jnk1* or *Jnk2* are fully viable, whereas mice with constitutive combined ablation of both *Jnk1* and *Jnk2* die during early development (4), suggesting a high degree of functional redundancy between both isoforms, warranting a combined ablation of *Jnk1* and *Jnk2* to investigate the role of this pathway by genetic means. Mice with combined conditional ablation of *Jnk1* and *Jnk2* in hepatocytes were generated by combining *Jnk1* floxed and *Jnk2* floxed mice with albumin-Cre mice (5). While these mice showed a clear phenotype in the mediation of liver steatosis when fed a high-fat diet, a spontaneous phenotype of mice with conditional ablation of *Jnk1* and *Jnk2* was not reported until the age of 24 wk (5). Therefore, in the present study, we examined the potential roles of JNK signaling during aging and unraveled a role of JNK signaling in the maintenance of hepatic and biliary homeostasis.

## Results

### Combined Ablation of *Jnk1* and *Jnk2* in Liver Parenchymal Cells Triggers Intrahepatic Cyst Development in Aging Mice.

To explore the hepatic function of the JNK pathway during aging, *Jnk1*^FL^ (floxed, ^FL^) mice and *Jnk2*^FL^ mice were interbred with *alfp*-cre mice to generate animals with conditional deletion of *Jnk1* (JNK1^LPC-KO^) (knockout, ^KO^), *Jnk2* (JNK2^LPC-KO^), or both *Jnk1* and *Jnk2* (JNK1/2^LPC-KO^) in liver parenchymal cells (LPCs) including hepatocytes and cholangiocytes, as JNK1/2^−/−^ mice are embryonically lethal (*SI Appendix*, Fig. S1 *A* and *B*) (6–9). Littermates carrying the respective loxP-flanked alleles but lacking expression of Cre recombinase were used as wild-type (WT) controls. We first examined JNK1^LPC-KO^ and JNK2^LPC-KO^ single-mutant mice at 6 and 52 wk of age and did not detect any phenotypic alterations at these time points (*SI Appendix*, Fig. S1 *C*–*F*). Macroscopic and microscopic analyses of the liver did not reveal alterations in hepatic architecture (*SI Appendix*, Fig. S1 *C* and *E*). Moreover, serological analyses in 6- and 52-wk-old mice did not reveal alterations in alanine aminotransferase (ALT) and glutamate dehydrogenase (GLDH) levels between JNK1^LPC-KO^ mice, JNK2^LPC-KO^ mice, and WT control mice. Of note, JNK1^LPC-KO^ mice showed slightly elevated alkaline phosphate (AP) levels at 6 wk of age, which were no longer altered at the age of 52 wk (*SI Appendix*, Fig. S1 *D* and *F*). AP levels of JNK2^LPC-KO^ mice were comparable to those of WT animals in both age groups (*SI Appendix*, Fig. S1 *D* and *F*).

**Fig. 1.**
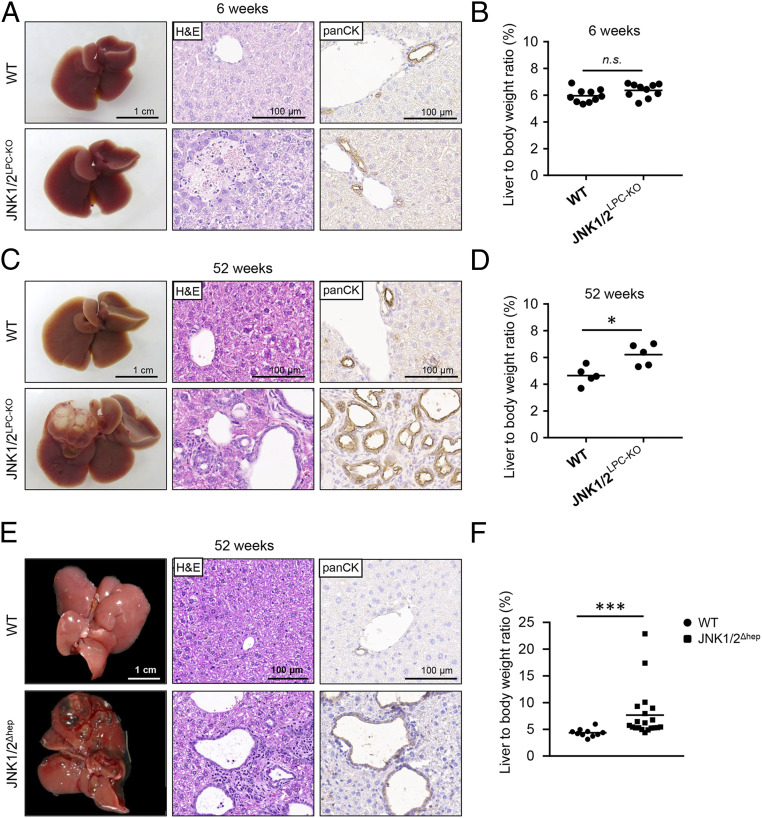
Combined deletion of *Jnk1*/*2* in LPCs induces massive liver cyst formation in old mice. (*A*) Representative macroscopic liver pictures (*Left*), hematoxylin and eosin (H&E) staining (*Middle*), and pan-CK staining (*Right*) of 6-wk-old WT and JNK1/2^LPC-KO^ animals. (*B*) Liver weight-to-body weight ratio of 6-wk-old WT and JNK1/2^LPC-KO^ animals. *n* = 10. (*C*) Macroscopic picture (*Left*), H&E staining (*Middle*), and pan-CK staining (*Right*) of 52-wk-old WT and JNK1/2^LPC-KO^ animals. (*D*) Liver weight-to-body weight ratio of 52-wk-old JNK1/2^LPC-KO^ and WT animals. *n* = 5. (*E*) Macroscopic picture (*Left*), H&E staining (*Middle*), and pan-CK staining (*Right*) of 52-wk-old JNK1/2^Δhep^ and WT animals. (*F*) Liver weight-to-body weight ratio of 52-wk-old JNK1/2^Δhep^ and WT animals. *n* = 10 to 20. n.s., not significant; *P < 0.05, ***P < 0.001.

Given the functional redundancy between JNK1 and JNK2, we next analyzed mice with combined ablation of *Jnk1* and *Jnk2* in LPCs. At the age of 6 wk, livers of JNK1/2^LPC-KO^ mice did not show macroscopic alterations and the liver weight-to-body weight ratios were comparable to WT animals (Fig. 1 *A* and *B*). Interestingly, microscopic analyses of the livers of JNK1/2^LPC-KO^ mice revealed the presence of necrotic areas, which had not been seen in single-*Jnk* mutant animals (Fig. 1*A*). In addition, these mice showed a profound change in liver architecture with increasing age. As such, at the age of 52 and 66 wk, JNK1/2^LPC-KO^ mice developed large multilocular biliary cysts, which were visible macroscopically and microscopically (Fig. 1*C* and *SI Appendix*, Fig. S2*A*). In addition, analysis of 52-wk-old JNK1/2^LPC-KO^ double-knockout mice showed a significantly increased liver weight-to-body weight ratio compared with WT mice (Fig. 1*D*). Histologically, cysts were lined by pan-cytokeratin–positive (pan-CK) biliary epithelial cells (Fig. 1*C* and *SI Appendix*, Fig. S2*A*), similar to cysts found in human (hereditary) fibropolycystic diseases of the liver, such as Caroli disease/syndrome (*SI Appendix*, Fig. S2*B*) (10–13).

A previous study analyzed the protein profile of cyst fluids from different human polycystic liver diseases by mass spectrometry and found a serum-like protein pattern (14). To determine the protein profile of the cyst found in JNK1/2^LPC-KO^ mice, we performed a mass spectrometry analysis, which also revealed a high content of proteins typical for serum samples like albumin, serotransferrin, complement 3, and various isoforms of α-1-antitrypsin (*SI Appendix*, Table S1 and Dataset S1). In addition, we could also detect unusually high levels of several nonserum proteins like clusterin, which is a predominantly secreted glycoprotein linked to processes like regulation of inflammation, lipid transport, apoptosis, and cell differentiation (15, 16).

**Fig. 2.**
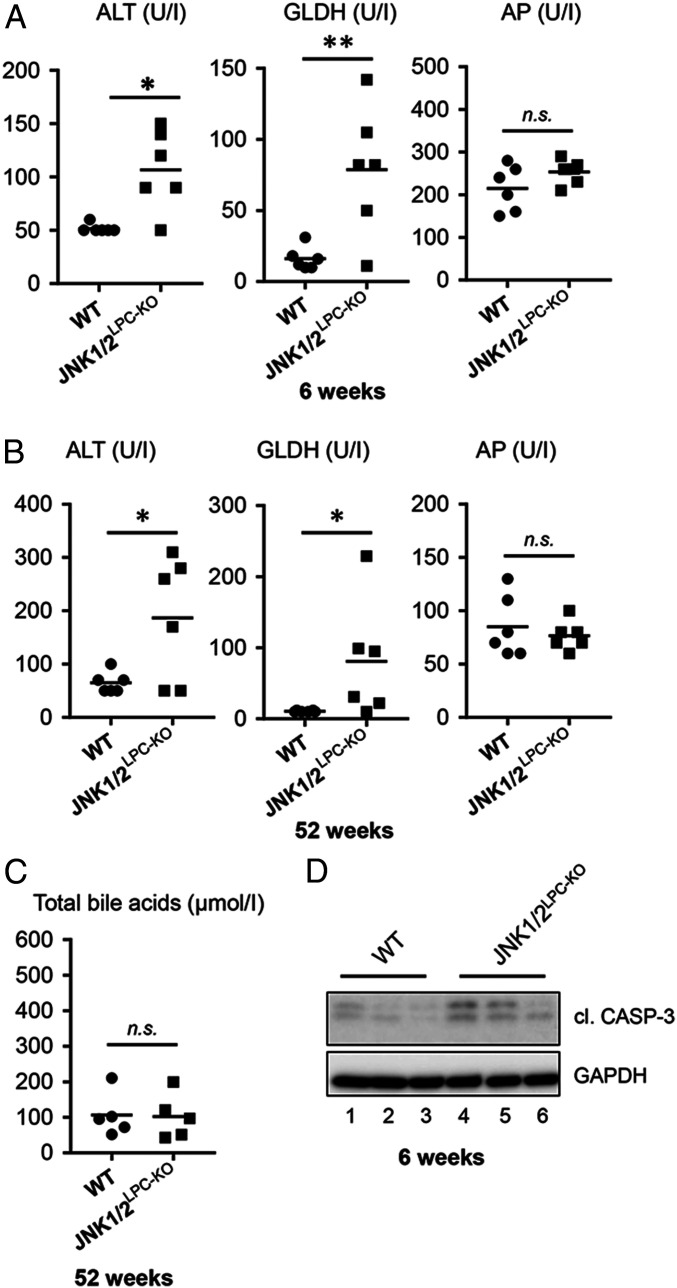
JNK1/2^LPC-KO^ mice show elevated markers for liver injury. (*A*) Serum-level analysis of ALT, GLDH, and AP in 6-wk-old WT and JNK1/2^LPC-KO^ mice. *n* = 6. (*B*) Serum-level analysis of ALT, GLDH, and AP in 52-wk-old WT and JNK1/2^LPC-KO^ mice. *n* = 6. (*C*) Serum-level analysis of total bile acids in 52-wk-old WT and JNK1/2^LPC-KO^ mice. *n* = 5. (*D*) Western blot analysis of liver protein extracts of 6-wk-old WT and JNK1/2^LPC-KO^ animals. *P < 0.05, **P < 0.01.

Conditional ablation of *Jnk1* and *Jnk2* in JNK1/2^LPC-KO^ mice was driven by an *alfp*-cre transgenic line, which mediates conditional ablation of floxed genes in hepatocytes, cholangiocytes, as well as hepatoblasts and precursor cells at embryonic day 10.5 (8, 17). To test if this unexpected cyst phenotype was independent of this specific cre line, we next regenerated conditional JNK1/2-knockout mice (JNK1/2^Δhep^) with an alternative cre line widely used in the field, namely the albumin-cre line, which targets the same albumin-expressing cell compartment as the *alfp* promotor (17) (*SI Appendix*, Fig. S2*C*). Macroscopic and microscopic analyses of the livers of JNK1/2^Δhep^ mice revealed that these mice also developed progressive biliary cysts with increasing age, which were clearly visible after 1 y (Fig. 1*E*). They also showed increased liver weight-to-body weight ratios (Fig. 1*F*), suggesting that the phenotype was not dependent on the alpha-feto-protein element activated in immature precursors of liver parenchymal cells in *alfp-cre* mice. An in situ hybridization in 52-wk-old Jnk1/2^Δhep^ mice also revealed that the deletion of Jnk remained stable throughout the aging process (*SI Appendix*, Fig. S2*D*). Importantly, we did not find any dysplastic changes in the progressed lesions in aged animals (up to 52 to 66 wk) nor invasive or infiltrative growth (Figs. 1 *C* and *E* and 2*B* and *SI Appendix*, Fig. S2*A*). Finally, to exclude that the cyst phenotype was caused by bacterial infection, we performed PCR for 16S ribosomal DNA in cyst tissue, which revealed no evidence of the presence of bacteria (*SI Appendix*, Fig. S2*E*).

Taken together, biliary cyst formation was observed in two distinct genetically modified mouse models with conditional hepatic ablation of *Jnk1*/*2*, showing that the phenotype occurred independent of the specific cre line used to target these floxed genes in parenchymal liver cells.

### Combined Deletion of *Jnk1* and *Jnk2* in LPCs Is Associated with Spontaneous Liver Injury.

Next, we aimed at analyzing the molecular mechanism underlying cyst development in mice with conditional ablation of *Jnk1* and *Jnk2* in parenchymal liver cells. JNK1/2^LPC-KO^ mice displayed areas of liver cell necrosis already at a young age, indicating the potential involvement of programmed cell death (PCD) in the mediation of the phenotype of JNK1/2-deficient livers (Fig. 1*A*) (18). Further, we measured serum levels of surrogate markers of liver and biliary injury in the distinct knockout models at 6 and 52 wk of age. This analysis revealed that ALT and GLDH levels were significantly increased in JNK1/2^LPC-KO^ mice at 6 wk of age and remained high at 52 wk of age, suggesting that hepatocyte cell death and potentially necrosis might be involved in cyst formation (Fig. 2 *A* and *B*). In contrast, AP as well as total bile acid levels as markers for cholestasis showed no significant elevation in JNK1/2^LPC-KO^ mice at 52 wk of age when compared with control animals, indicating a functional biliary system in JNK1/2^LPC-KO^ mice up to this age (Fig. 2 *A*–*C*).

To further examine if—beyond the observed morphological signs of spontaneous necrosis in young JNK1/2^LPC-KO^ mice (Fig. 1*A*)—also apoptosis could be involved in the mediation of liver injury, we performed Western blot analysis for CASPASE-3 cleavage. This analysis revealed slightly increased levels of the cleaved form of CASPASE-3 in 6-wk-old JNK1/2^LPC-KO^ mice (Fig. 2*D*). Together, these findings provided evidence that the development of biliary cysts in JNK1/2^LPC-KO^ mice was associated with spontaneous necrosis and apoptosis of parenchymal liver cells.

### Cyst Formation in JNK1/2^LPC-KO^ Mice Is Not Rescued by Genetic Inhibition of Apoptosis or Necroptosis.

**Fig. 3.**
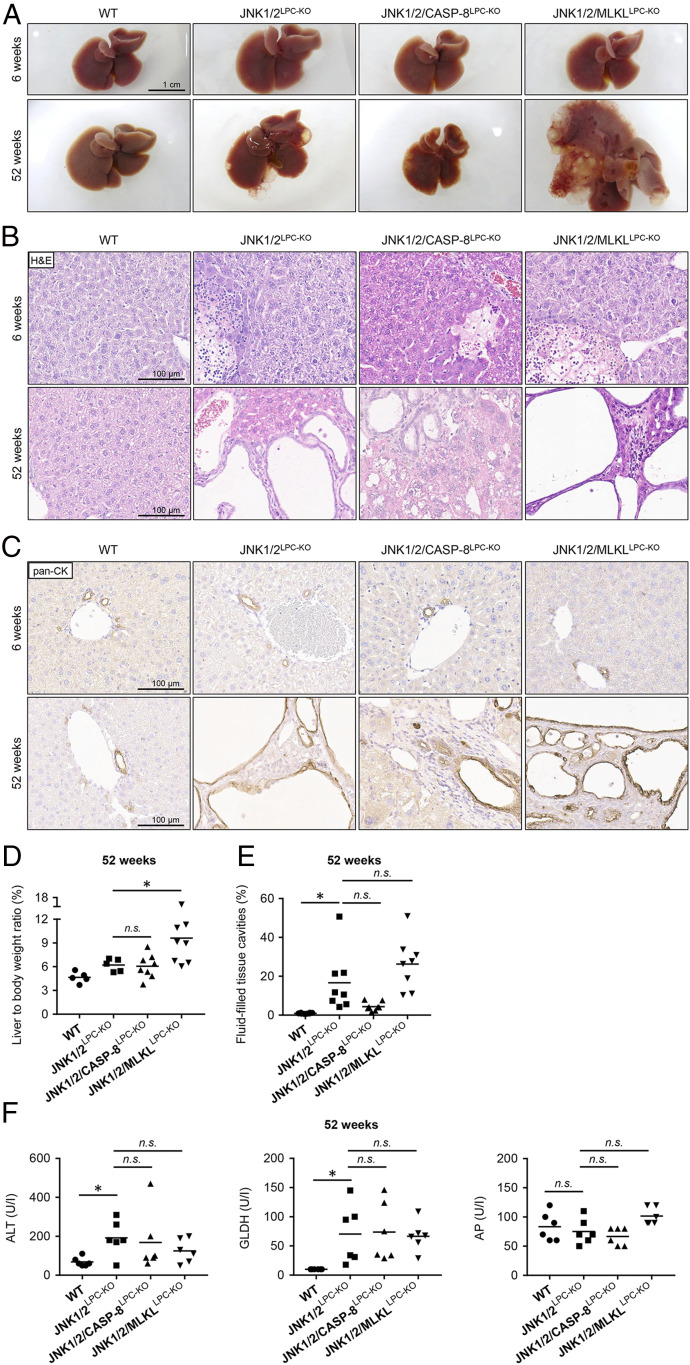
Additional deletion of *Caspase-8* or *Mlkl* does not significantly reduce cyst formation in JNK1/2^LPC-KO^ mice. (*A*) Representative macroscopic liver pictures of WT (*Left*), JNK1/2^LPC-KO^ (*Middle Left*), JNK1/2/CASP-8^LPC-KO^ (*Middle Right*), and JNK1/2/MLKL^LPC-KO^ (*Right*) animals at the age of 6 wk (*Top*) and 52 wk (*Bottom*). (*B*) Representative H&E-stained liver sections of WT (*Left*), JNK1/2^LPC-KO^ (*Middle Left*), JNK1/2/CASP-8^LPC-KO^ (*Middle Right*), and JNK1/2/MLKL^LPC-KO^ (*Right*) animals at the age of 6 wk (*Top*) and 52 wk (*Bottom*). (*C*) Representative pan-CK–stained liver sections of WT (*Left*), JNK1/2^LPC-KO^ (*Middle Left*), JNK1/2/CASP-8^LPC-KO^ (*Middle Right*), and JNK1/2/MLKL^LPC-KO^ (*Right*) animals at the age of 6 wk (*Top*) and 52 wk (*Bottom*). (*D*) Liver weight-to-body weight ratio of 52-wk-old WT, JNK1/2^LPC-KO^, JNK1/2/CASP-8^LPC-KO^, and JNK1/2/MLKL^LPC-KO^ animals. *n* = 5 to 8. (*E*) Quantification of fluid-filled tissue cavities, including liver cysts, biliary ducts, and blood vessels, of 52-wk-old WT, JNK1/2^LPC-KO^, JNK1/2/CASP-8^LPC-KO^, and JNK1/2/MLKL^LPC-KO^ mice. *n* = 6 to 8. (*F*) Serum analysis of ALT, GLDH, and AP in 52-wk-old WT, JNK1/2^LPC-KO^, JNK1/2/CASP-8^LPC-KO^, and JNK1/2/MLKL^LPC-KO^ mice. *n* = 6. *P < 0.05.

In order to further dissect the molecular mechanism of biliary cyst development in Jnk1/2^LPC-KO^ mice, we evaluated if blockage of either apoptosis or necroptosis might ameliorate this dramatic phenotype or might even rescue Jnk double-mutant mice from cyst development. To test this, we generated mice with combined deletion of *Jnk1*/*2* together with either *Caspase-8* (JNK1/2/CASP-8^LPC-KO^) [blockage of apoptosis (19)] or *Mlkl* (JNK1/2/MLKL^LPC-KO^) (blockage of necroptosis) in LPCs (*SI Appendix*, Fig. S3*A*). Analysis of JNK1/2/CASP-8^LPC-KO^ mice showed that the additional deletion of *Caspase-8* did not rescue cyst formation at 52 wk of age (Fig. 3 *A*–*C*). Histological and immunohistochemical analyses confirmed the presence of biliary cysts (macroscopically and microscopically visible) and also necrotic areas in JNK1/2/CASP-8^LPC-KO^ mice (Fig. 3 *B* and *C*). Further analysis of the liver weight-to-body weight ratios and additional quantification of the fluid-filled tissue cavities revealed no significant differences between JNK1/2^LPC-KO^ and JNK1/2/CASP-8^LPC-KO^ mice (Fig. 3 *C* and *D*).

Since the inhibition of apoptosis in JNK1/2^LPC-KO^ mice did not lead to a rescue of the liver cyst phenotype, we next examined the function of necroptosis in this model. Additional ablation of *Mlkl* in JNK1/2^LPC-KO^ mice did not rescue from cyst development either and showed pronounced cyst formation in 52-wk-old mice (Fig. 3 *A*–*C*). Analysis confirmed a significant increase in liver weight-to-body weight ratios (Fig. 3*D*), while quantification of fluid-filled spaces showed no significant increase between JNK1/2^LPC-KO^ and JNK1/2/MLKL^LPC-KO^ mice (Fig. 3*E*). Of note, the presumable necrotic areas remained present in 6-wk-old JNK1/2/MLKL^LPC-KO^ animals (Fig. 3*B* and *SI Appendix*, Fig. S3*B*), arguing that these necrotic areas did not reflect pure necroptosis. Neither additional deletion of *Caspase-8* nor additional deletion of *Mlkl* led to a significant change in serum levels of ALT and GLDH (Fig. 3*F*).

### Cyst Development in JNK1/2^LPC-KO^ Mice Is Mediated by a CASPASE-8–Related Function of RIPK1.

Based on the finding that—despite no changes in the overall degree of spontaneous liver injury measured by serum levels of liver enzymes like ALT—hepatic cyst development was not substantially influenced by genetic modifications of either *Caspase-8* or *Mlkl* alone, we hypothesized that targeting simultaneously both cell-death pathways through additional deletion of *Ripk1* in JNK1/2^LPC-KO^ mice might rescue cyst formation (*SI Appendix*, Fig. S4*A*). Receptor-interacting protein kinase 1 (RIPK1) is a central molecule in the regulation of survival, apoptosis, and necroptosis and an important interaction partner of CASPASE-8 as well as mixed lineage kinase domain-like pseudokinase (MLKL) (20–24). In line with this hypothesis, we found RIPK1 expression up-regulated in cystic areas of JNK1/2^LPC-KO^ mice (*SI Appendix*, Fig. S4*B*).

**Fig. 4.**
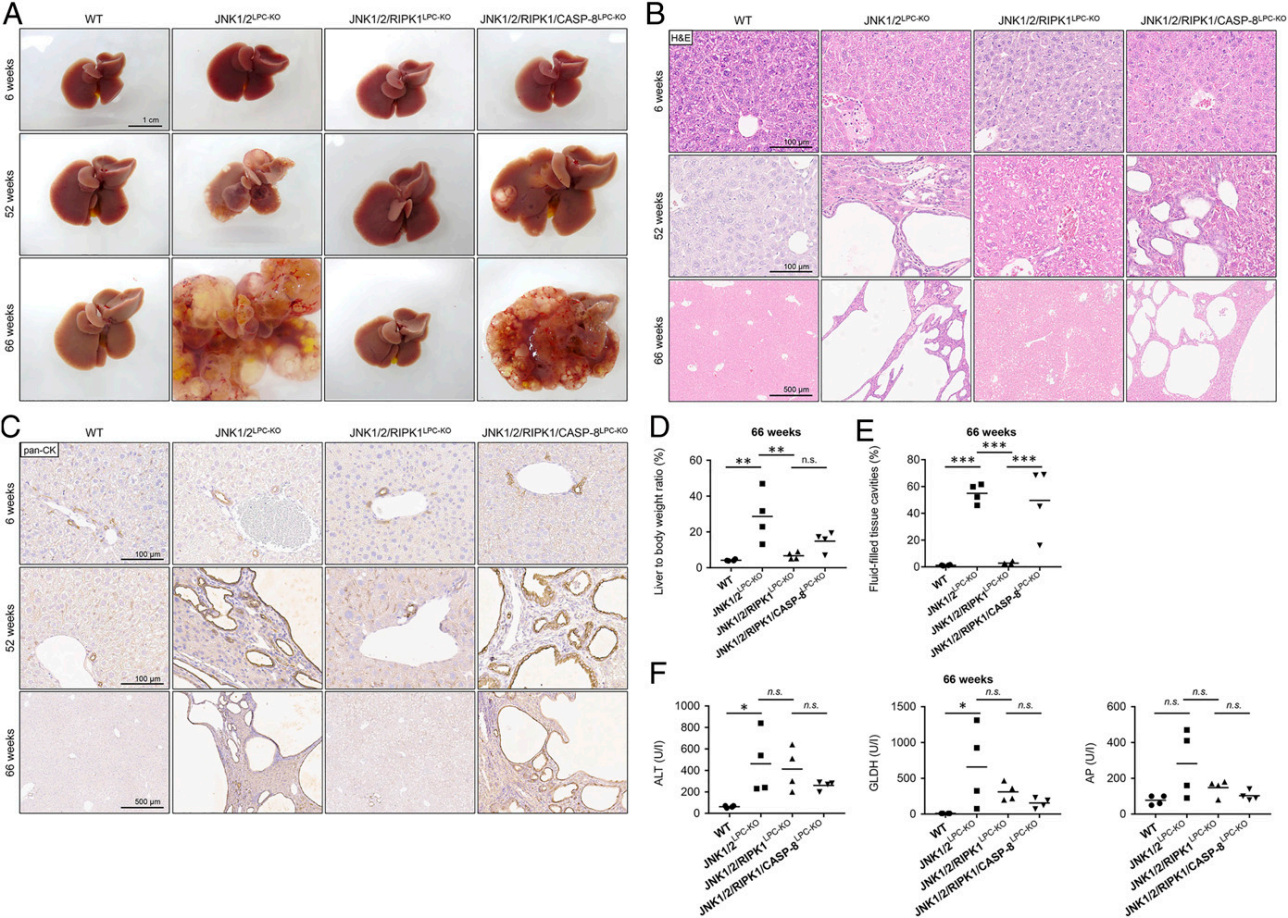
*Ripk1* deletion rescues JNK1/2^LPC-KO^ mice from macroscopic visible cyst formation, which is restored by additional deletion of *Caspase-8*. (*A*) Representative macroscopic pictures of 6-wk- (*Top*), 52-wk- (*Middle*), and 66-wk- (*Bottom*) old WT (*Left*), JNK1/2^LPC-KO^ (*Middle Left*), JNK1/2/RIPK1^LPC-KO^ (*Middle Right*), and JNK1/2/RIPK1/CASP-8^LPC-KO^ (*Right*) livers. (*B*) Representative H&E stainings of liver sections of 6-wk- (*Top*), 52-wk- (*Middle*), and 66-wk- (*Bottom*) old WT (*Left*), JNK1/2^LPC-KO^ (*Middle Left*), JNK1/2/RIPK1^LPC-KO^ (*Middle Right*), and JNK1/2/RIPK1/CASP-8^LPC-KO^ (*Right*) mice. (*C*) Representative pan-CK stainings of liver sections of 6-wk- (*Top*), 52-wk- (*Middle*), and 66-wk- (*Bottom*) old WT (*Left*), JNK1/2^LPC-KO^ (*Middle Left*), JNK1/2/RIPK1^LPC-KO^ (*Middle Right*), and JNK1/2/RIPK1/CASP-8^LPC-KO^ (*Right*) mice. (*D*) Liver weight-to-body weight ratios of 66-wk-old WT, JNK1/2^LPC-KO^, JNK1/2/RIPK1^LPC-KO^, and JNK1/2/RIPK1/CASP-8^LPC-KO^ mice. *n* = 4. (*E*) Quantification of fluid-filled tissue cavities, including liver cysts, biliary ducts, and blood vessels, of 66-wk-old WT, JNK1/2^LPC-KO^, JNK1/2/RIPK1^LPC-KO^, and JNK1/2/RIPK1/CASP-8^LPC-KO^ mice. *n* = 4. (*F*) Serum analysis of ALT, GLDH, and AP in 66-wk-old WT, JNK1/2^LPC-KO^, JNK1/2/RIPK1^LPC-KO^, and JNK1/2/RIPK1/CASP-8^LPC-KO^ mice. *n* = 4. *P < 0.05, **P < 0.005, ***P < 0.001.

Strikingly, additional deletion of *Ripk1* prevented the development of macroscopically visible cysts in JNK1/2^LPC-KO^ mice and almost completely rescued the biliary phenotype at the microscopic level (Fig. 4 *A*–*C*). In line with this, quantification of liver weight-to-body weight ratio and the area of fluid-filled cavities revealed significant reduction upon ablation of *Ripk1* (Fig. 4 *D* and *E*). Interestingly, despite the rescue from cyst development, additional deletion of *Ripk1* did not significantly alter ALT, GLDH, and AP serum levels (Fig. 4*F* and *SI Appendix*, Fig. S4*C*) but led to a significant reduction of the necrotic areas compared with JNK1/2^LPC-KO^ animals, suggesting ameliorated but ongoing hepatocellular damage (*SI Appendix*, Fig. S4*D*).

We have previously shown that hepatocytic RIPK1 not only mediates cell death through its kinase activity but is also capable of inhibiting CASPASE-8–dependent apoptosis through a kinase-independent scaffolding function (24). In order to test the functional interaction of RIPK1 with CASPASE-8 in cyst formation of JNK1/2^LPC-KO^ mice, we intercrossed JNK1/2/RIPK1^LPC-KO^ mice with *Caspase-8*^FL^ mice (JNK1/2/RIPK1/CASP-8^LPC-KO^) (*SI Appendix*, Fig. S4*A*). Strikingly, the massive cyst phenotype, which was lost in JNK1/2/RIPK1^LPC-KO^ mice, was fully restored in quadruple-knockout mice with an additional loss of *Caspase-8* (Fig. 4 *A*–*E*). Of note, CASP-8^LPC-KO^ mice did not show any signs of liver cyst formation with increasing age (*SI Appendix*, Fig. S4*E*). Additional deletion of *Caspase-8* in JNK1/2/RIPK1^LPC-KO^ mice did not lead to a reinduction of the formation of necrotic areas, which again argues for divergent mechanisms leading to both a hepatocellular and biliary phenotype in JNK1/2^LPC-KO^ mice (*SI Appendix*, Fig. S4*D*).

**Fig. 5.**
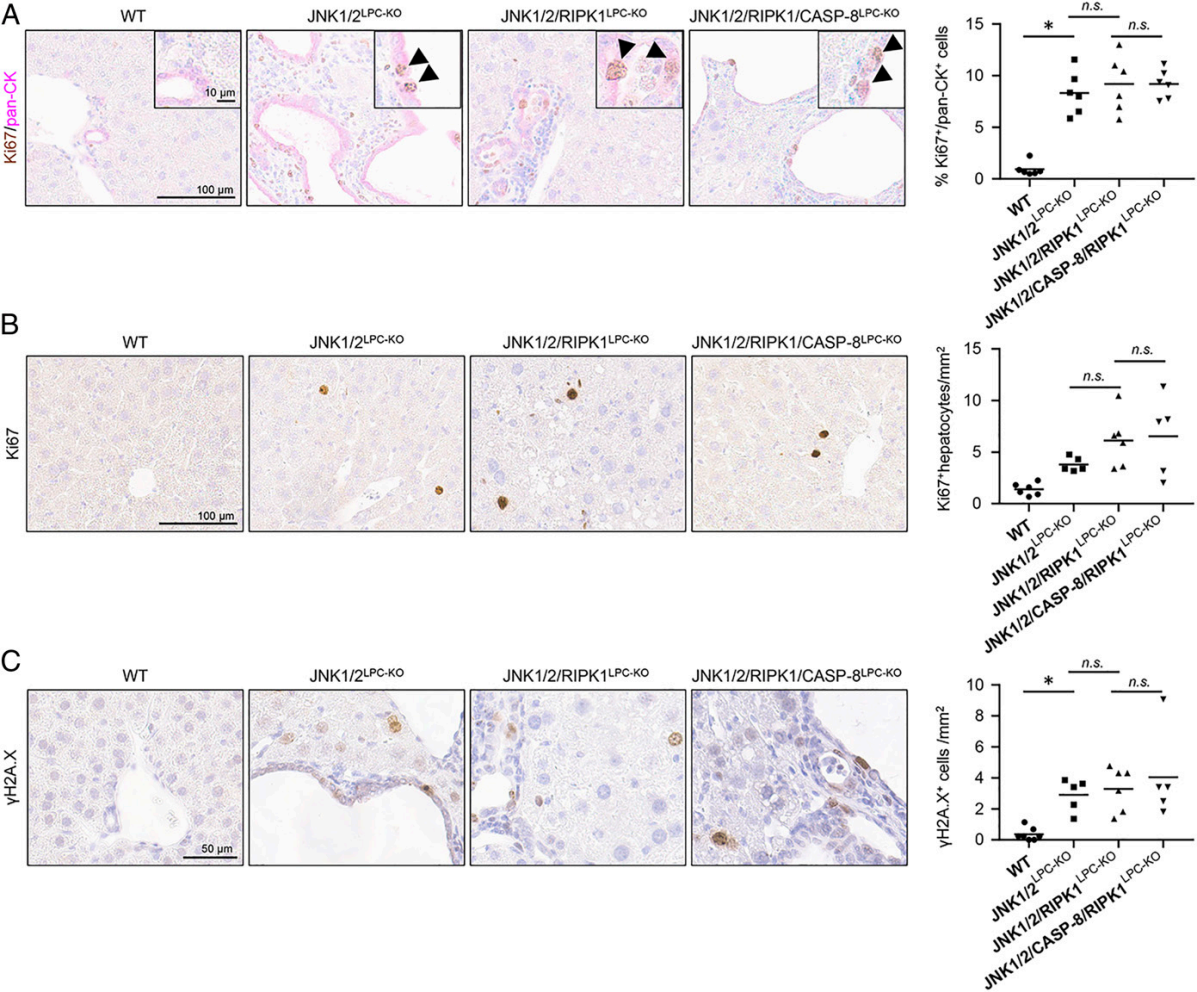
Additional deletion of *Ripk1* in JNK1/2^LPC-KO^ does not influence proliferation and DNA-damage response in cystic liver tissue. (*A*) Representative Ki67 (brown) and pan-CK (red) double stainings of liver sections of 52-wk-old WT, JNK1/2^LPC-KO^, JNK1/2/RIPK1^LPC-KO^, and JNK1/2/RIPK1/CASP-8^LPC-KO^ mice and quantification of double-positive cells. *n* = 6. Arrowheads indicate double-positive cells. (*B*) Representative Ki67 stainings of liver sections of 52-wk-old WT, JNK1/2^LPC-KO^, JNK1/2/RIPK1^LPC-KO^, and JNK1/2/RIPK1/CASP-8^LPC-KO^ mice and quantification. *n* = 5 or 6. (*C*) Representative ɣH2A.X stainings of liver sections of 52-wk-old WT, JNK1/2^LPC-KO^, JNK1/2/RIPK1^LPC-KO^, and JNK1/2/RIPK1/CASP-8^LPC-KO^ mice and quantification. *n* = 5 to 7. *P < 0.05.

RIPK1 and CASPASE-8 were previously shown to have a cell death-independent function in the regulation of DNA-damage responses and compensatory proliferation as the basis of cancer development in the liver (25). To test if this respective pathway was specifically altered upon additional ablation of *Ripk1* and *Caspase-8*, we performed immunohistochemical analysis of liver tissues from JNK1/2^LPC-KO^, JNK1/2/RIPK1^LPC-KO^, and JNK1/2/RIPK1/CASP-8^LPC-KO^ mice for markers of proliferation (Ki67) and DNA-damage responses (phosphorylation of histone H2A.X on serine 139 [ɣH2A.X]). Additional ablation of *Ripk1* and *Caspase-8* did not affect numbers of proliferating cholangiocytes (Fig. 5*A*) and hepatocytes (Fig. 5*B*) and also did not change numbers in ɣH2A.X-positive LPCs (Fig. 5*C*) compared with JNK1/2^LPC-KO^ mice. This argues against a role of DNA-damage response in the RIPK1/CASPASE-8–dependent mediation of the cyst phenotype in JNK1/2^LPC-KO^ mice, suggesting that a cell-death function of RIPK1 may be responsible for the phenotype. This function might occur eventually on a single-cell basis, which is not reflected by conventional molecular analyses.

### Cyst Formation in JNK1/2^LPC-KO^ Mice Primarily Originates from Cholangiocytes.

**Fig. 6.**
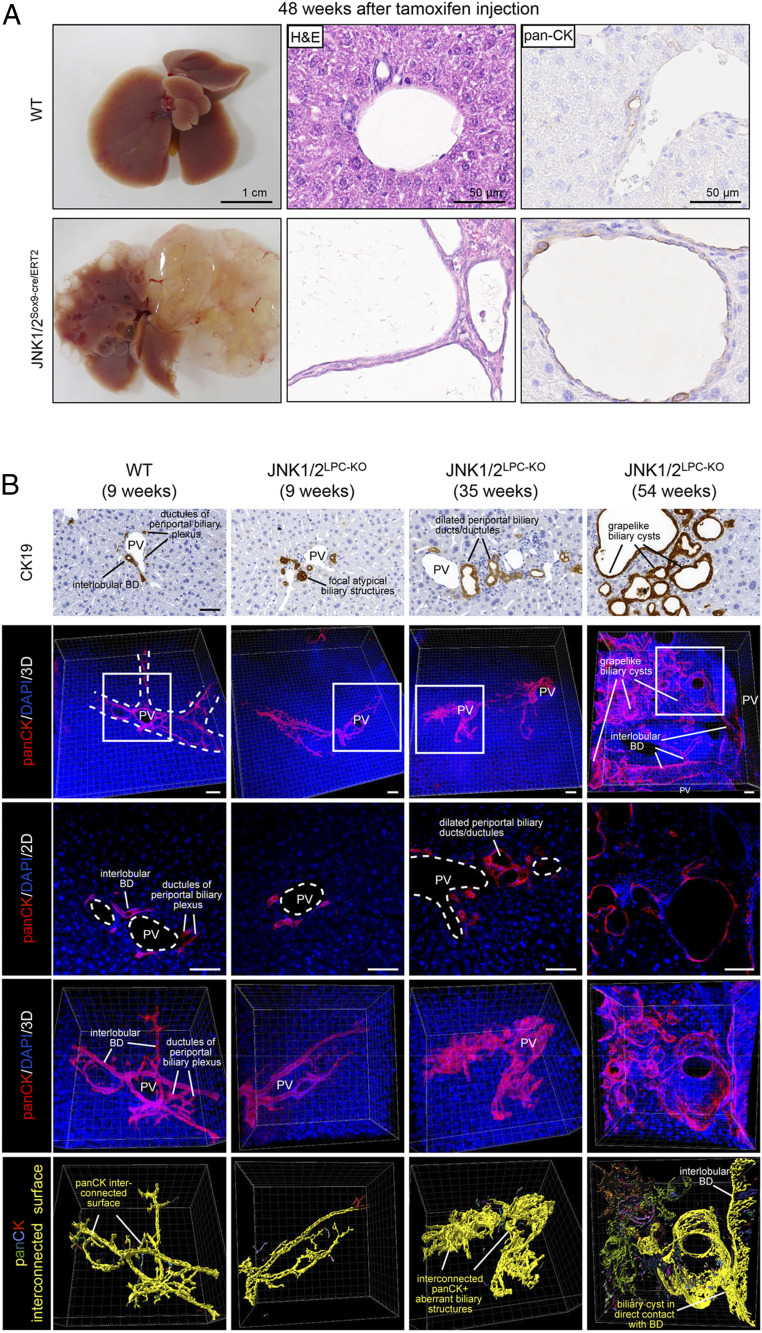
Liver cysts in JNK1/2 ablated livers originate from bile duct cells. (*A*) Representative macroscopic liver picture (*Left*), H&E staining (*Middle*), and pan-CK staining (*Right*) of 52-wk-old WT and JNK1/2^Sox9-cre/ERT2^ animals 48 wk after tamoxifen injection. (*B*) Stepwise cystic transformation of intrahepatic bile ducts in JNK1/2^LPC-KO^ animals. Representative CK19 immunohistochemistry stainings (first row) and three-dimensional (3D) analysis of pan-CK/DAPI–immunostained intrahepatic bile ducts/ductules showing progressive stepwise cystic transformation of intrahepatic bile duct/ductules in JNK1/2^LPC-KO^ animals at the indicated age (low-magnification 3D maximum-intensity projection, second row; corresponding optical 2D view and 3D maximum-intensity projection of enlarged insets, third and fourth rows; corresponding 3D pan-CK + surface/interconnectivity analysis using the IMARIS surface protocol where different colors represent individual interconnected panCK+ segments, fifth row). White dashed lines, portal tract; white dashed circles, portal vein; BD, bile duct; PV, portal vein. (Scale bars, 50 µm.)

We finally aimed at further dissecting the cell type of origin for cyst development in JNK1/2^LPC-KO^ mice. For this, we generated a mouse line with an inducible deletion of JNK1/2 primarily in the biliary cell compartment using the Sox9-cre/ERT2 mouse line, which primarily targets the biliary cell compartment and additionally few single periportal hepatocytes (26). Of note, JNK1/2^Sox9-cre/ERT2^ mice developed massive macroscopically and microscopically visible cysts, which resembled the phenotype of JNK1/2^LPC-KO^ mice, 48 wk after tamoxifen injection (Fig. 6*A*). This finding provided evidence that the phenotype in JNK1/2^LPC-KO^ mice arose dominantly from the biliary cell compartment.

To further substantiate this hypothesis, we performed a three-dimensional imaging analysis of the liver cysts in JNK1/2^LPC-KO^ mice based on the reconstruction of optical serial immunofluorescence images to get a comprehensive understanding of the liver cyst origin, architecture, and connection to the biliary network (Fig. 6*B*). This interconnectivity analysis identified the cysts as focally dilated duct structures, which most likely originated from the intrahepatic ducts and ductules, whereas biliary metaplastic hepatocytes were not detected. An additional “surface reconstruction” showed a progressive stepwise cystic transformation of intrahepatic bile ducts in JNK1/2^LPC-KO^ animals at the indicated ages and a morphological connection of these dilated structures, providing further morphological evidence that the cysts in JNK1/2^LPC-KO^, JNK1/2^Δhep^, and JNK1/2^Sox9-cre/ERT2^ mice arose from the biliary system itself rather than from, for example, transdifferentiated hepatocytes.

To further analyze the potential function of RIPK1 in *Jnk1*/*2*-deficient cholangiocytes, we performed a kinase activity-profiling microarray focusing on threonine/serine kinases. This array analyzes 144 peptide substrates with known phosphorylation sites. For this, primary mouse cholangiocytes were pretreated with JNK inhibitor (SP600125) or solvent (dimethyl sulfoxide, DMSO) and additionally stimulated with TNF for 1 h. The kinase activity-profiling microarray was performed using isolated protein extracts. We focused on the effect of TNF stimulation in the two different conditions (solvent or JNK inhibition) and analyzed the specific kinase activity profiles (*SI Appendix*, Figs. S5 and S6). Compared with solvent treatment (*SI Appendix*, Fig. S5), inhibition of JNK resulted in a specifically altered activation pattern, implicating a compensatory signal cascade activation upon JNK inhibition (*SI Appendix*, Fig. S6). A further kinase interaction network analysis in relation to RIPK1 revealed the activation of several different kinases, including the p38α substrates MK2 (MAPKAPK2) and MK3 (MAPKAPK3), which did not appear in the activity profile of solvent-treated cells (*SI Appendix*, Figs. S7 and S8). Of note, it was previously suggested that deletion or inhibition of MAPKs can lead to compensatory hyperactivation of other MAPKs. As such, deletion of p38α leads to hyperactivation of JNK in hepatocytes (27). Moreover, previous studies suggested that MK2 can phosphorylate RIPK1 in response to proinflammatory stimuli, such as TNF, thereby modulating its activity (28). Therefore, these data suggest a molecular link between JNK deletion/inhibition, MK2 and/or MK3 activation, and biliary cyst formation through the modulation of RIPK1 activity.

### RIPK1 Expression Is Found in a Subset of Patients with Polycystic Liver Disease.

**Fig. 7.**
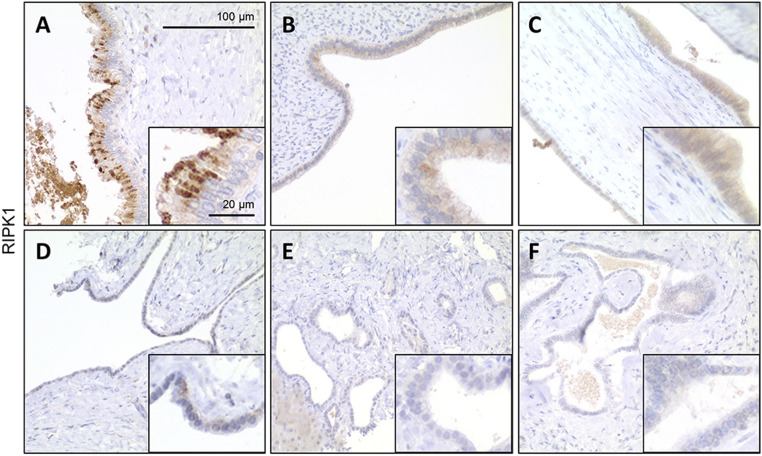
Human liver samples of Caroli disease show expression of RIPK1 in biliary cystic epithelium. (*A*) Cystic bile duct in Caroli disease showing strong patchy RIPK1 staining in biliary epithelium. (*B*) Another case of Caroli disease with weak RIPK1 expression in some biliary epithelial cells lining the cystically dilated bile duct. (*C*) Focal RIPK1 staining in Caroli syndrome. Note the lack of RIPK1 staining in a corresponding ductal plate malformation of this patient (*F*). (*D*) Barely visible RIPK1 immunosignal in this example of autosomal dominant polycystic liver disease. (*E*) No expression in this example of autosomal recessive polycystic liver disease.

We finally aimed at providing evidence for a potential relevance of RIPK1 in the development of human (hereditary) fibropolycystic diseases of the liver. For this, we evaluated the protein levels of RIPK1 in human liver samples of various entities belonging to the group of (hereditary) fibropolycystic diseases of the liver by immunohistochemistry. As shown in Fig. 7, RIPK1 was consistently detectable in biliary epithelial cells of cystically dilated bile ducts of patients with Caroli disease/syndrome (with additional congenital hepatic fibrosis) (Fig. 7 *A*–*C* and *F* and *SI Appendix*, Table S2). In contrast, this was not seen in patients with autosomal dominant or autosomal recessive variants of polycystic disease and biliary microhamartoma (von Meyenburg complex) (Fig. 7 *D* and *E* and *SI Appendix*, Table S2), suggesting that RIPK1 might play a specific role in the pathogenesis of hepatic cyst formation, as it does in mice with JNK1/2-deficient cholangiocytes.

## Discussion

Stress-activated kinases like JNKs play a crucial role in various physiological and pathophysiological processes such as proliferation, invasive migration, therapy resistance, and PCD (18). However, like many other signaling pathways, their specific function in the liver in mouse disease models was previously studied using young mice (5). Compared with humans, this translates to the situation in adolescents, while many liver diseases such as metabolic diseases typically manifest in elderly people. Hence, liver diseases of the elderly are often not adequately represented by experimental models (29), suggesting that more attention needs to be drawn to phenotypic changes and molecular alterations during aging. Here, we investigated the hepatic function of JNK1/2 and could show a function of JNK1/2, RIPK1, and CASPASE-8 in the maintenance of biliary homeostasis during aging.

Biliary cyst formation is found in up to 10% of the general population and up to 94% of patients are older than 35 y of age. Several diseases associated with biliary cysts belong to the group of (hereditary) fibropolycystic diseases of the liver, characterized by the development of several large cysts and a broad range of severity (30). Entities belonging to this disease spectrum include autosomal dominant and autosomal recessive polycystic (30, 31) Caroli disease/syndrome as well as biliary microhamartoma (von Meyenburg complex). Caroli disease represents a rare congenital disease with cystic dilation of the bile ducts leading to congenital cysts of the intrahepatic bile ducts (32). Complications are related to impaired bile drainage, providing the basis for recurrent cholangitis and progressive liver fibrosis leading to portal hypertension, which may require liver transplantation (32, 33). The molecular mechanisms underlying (hereditary) fibropolycystic diseases of the liver are still incompletely understood, although germline mutations have been identified which are associated with specific forms such as mutations of the genes *Pkd1* and *Pkd2* in autosomal dominant polycystic kidney disease (ADPKD) (30, 32, 33).

Previous studies linked JNKs to PKD, but the function of JNK signaling in these diseases was not clearly defined. An early study showed that silencing of polycystin-1 (the protein product of *Pkd1*) enhanced thrombin-induced apoptosis, JNK activation, and BCL-2 degradation in Madin–Darby canine kidney cells (34). Furthermore, analysis of *cpk* mice, which are used as a mouse model of PKD and carry a mutation of the gene *Cys1* (Cystin-1), also revealed an increase of JNK phosphorylation in the cystic kidney tissue (35). In contrast, DBA/2-*pcy*/*pcy* mice—another PKD mouse model—showed a down-regulation of JNK phosphorylation in the cyst epithelium (36). However, these studies were focused on kidney cysts. We are not aware of data focusing on the role of JNK-dependent signaling in (hereditary) fibropolycystic diseases of the liver.

In this study, we found that deletion of *Jnk1* and *Jnk2* in LPCs resulted in massive cyst formation in aging mice. We identified these cysts as focal dilated duct structures which were morphologically connected and originated from the intrahepatic bile duct system. Of note, we could not detect neoplastic or dysplastic areas, arguing against the development of an intrahepatic cholangiocarcinoma (iCCA) or any iCCA precursor lesions (37). The cyst fluid of JNK1/2^LPC-KO^ mice contained a serum-like protein pattern, which is similar to a protein profile found in human biliary cysts (14). In addition, we could detect a high expression of different proteins such as clusterin, which is linked to various physiological processes such as apoptosis and cell differentiation (15, 16). Clusterin has also been identified as a component of renal cyst fluid in human autosomal recessive polycystic kidney disease (ARPKD) patients (38). Up to now, no connection between polycystic liver disease and clusterin expression is known. Some studies suggested a role of clusterin in liver cancer development (39, 40). Of note, JNK1/2^LPC-KO^ and JNK1/2^Δhep^ mice in our study showed no evidence of hepatocellular carcinoma or cholangiocarcinoma up to the age of 52 wk.

Our findings indicated the presence of liver injury already in young JNK1/2^LPC-KO^ mice, which was associated with spontaneous apoptosis as well as necrosis. This observation prompted us to specifically evaluate the potential function of molecules known to regulate PCD in the development of biliary cysts in JNK1/2^LPC-KO^ mice. While the additional deletion of *Caspase-8* or *Mlkl* alone did not rescue the cyst phenotype in JNK1/2^LPC-KO^, additional deletion of *Ripk1* prevented biliary cyst formation (Figs. 3 and 4). At present, the exact nature of this functional interaction is not clear. While JNK1/2^LPC-KO^ mice showed liver injury to a certain degree, we did not detect a significant alteration of overall liver injury measured by serum enzymes like ALT, GLDH, or AP when we additionally deleted Mlkl (blockage of necroptosis; tendency to more cyst development), Caspase-8 [blockage of apoptosis and potential activation of necroptosis (19); tendency to less cyst development], or Ripk1 [inhibition of necroptosis/potential activation of apoptosis (24); rescue from cyst development] (Fig. 4). This finding appears counterintuitive at first glance, but could have two alternative functional explanations.

First, RIPK1 and CASPASE-8 could exert cell death-independent functions in JNK1/2^LPC-KO^ mice to promote biliary cyst formation. As such, previous studies including our own revealed that CASPASE-8 and RIPK1 ensure tissue homeostasis by forming signaling complexes which can sense DNA damage or control chromosome segregation during mitosis, a function that might be relevant in the biliary cyst phenotype in JNK1/2^LPC-KO^ mice. A disruption of complex formation, be it through knockout or pharmacological inhibition of CASPASE-8 or RIPK1, resulted in genetic/chromosomal instability that could trigger carcinogenesis (21, 25). This respective DNA damage-sensing mechanism acted via JNK and resulted in the phosphorylation of histone H2A.X (γH2A.X). However, while analysis of 52-wk-old JNK1/2^LPC-KO^ mice revealed a moderately increased level of γH2A.X, this level was not affected by the additional deletion of *Ripk1* or *Ripk1*/*Caspase-8*, arguing against the involvement of this respective RIPK1/CASPASE-8–dependent DNA-damage response pathway in biliary cyst formation of JNK1/2^LPC-KO^ mice. Recently, a direct proproliferative function of RIPK1 was suggested in CCA. Of note, it was suggested that this respective function does not only depend on p38, ERK1/2, and AP-1 but also on the presence of JNK (41). To exclude a proliferation-promoting function of RIPK1 as a crucial element for cyst formation, we examined the level of proliferation in the liver tissue of the different KO mice. Remarkably, we could not detect a significant difference in epithelial cell proliferation between JNK1/2^LPC-KO^, JNK1/2/RIPK1^LPC-KO^, and JNK1/2/RIPK1/Casp-8^LPC-KO^ mice, arguing against a direct RIPK1-dependent modulation of biliary cell proliferation as the reason for liver cyst development in JNK1/2^LPC-KO^ mice.

Alternatively, our data provided evidence that in the absence of JNK1 and JNK2, RIPK1 could transmit a survival signal in cholangiocytes that becomes relevant upon aging and thus might counteract single cellular cell-death events triggered through aging-induced cellular stress. It cannot be fully excluded that JNKs directly mediate phosphorylation of RIPK1 and change its activation status. However, our kinase activity-profiling microarray in primary cholangiocytes provided evidence that the p38α substrate MK2—a known RIPK1-interacting kinase—was activated upon JNK inhibition, which might be triggered through upstream MAPKs capable of phosphorylating both JNKs and p38α and redirect their kinase activity in the absence of one substrate. MK2 in turn is known to phosphorylate RIPK1 in response to proinflammatory stimuli, such as TNF, thereby inhibiting RIPK1 autophosphorylation and suppressing RIPK1-dependent apoptosis and necroptosis (28). Presumably, such single or multiple inhibitory phosphorylations of RIPK1 could inhibit cell death in cholangiocytes, which in turn could promote the expansion of these cysts in JNK1/2^LPC-KO^ mice. Therefore, the additional ablation of *Ripk1*, a genetic event known to trigger cellular apoptosis (24), would remove this cell-death blockade and lead to the net reduction of biliary cells observed in JNK1/2/RIPK1^LPC-KO^ mice. In line with this, additional *Caspase-8* deletion and concomitant apoptosis inhibition restored cyst formation (JNK1/2/RIPK1/Casp-8^LPC-KO^ mice). This would also explain why single *Caspase-8* or *Mlkl* ablation did not prevent the cyst phenotype seen in JNK1/2^LPC-KO^ mice. The lack of evidence for the increased cell-death induction in JNK1/2/RIPK1^LPC-KO^ mice probably points to single-cell events that are hardly detectable by conventional molecular methods, as they might occur at specific time points. Importantly, our kinase array data provided no functional proof of this hypothesis and we cannot exclude that other molecular linkages between JNKs and RIPK1 exist in cholangiocytes. However, several recent publications showed that kinases such as IKKα, IKKβ, and TAK1 can phosphorylate RIPK1 via MK2, thereby inhibiting spontaneous apoptosis and necroptosis activation in liver cells (23, 42, 43). In line with this, genetic ablation of Ikkα/β or Tak1 in LPCs inhibited biliary proliferation and resulted in biliary paucity (23, 44, 45), while Jnk deletion resulted in expansion of biliary cells in these mice.

Together, our data suggest a functional concept of how stress-related kinases in general and JNK specifically interact with the PCD mediator RIPK1 in physiology and pathophysiology. A deeper functional understanding of the cell type-specific functions of this JNK1/2/RIPK1/CASPASE-8–dependent signaling module could lead to a better understanding of biliary regeneration and cellular plasticity in the liver and enable the development of novel therapeutic strategies in polycystic liver diseases.

## Materials and Methods

### Generation of Genetically Modified Mouse Models.

Mice carrying loxP site-flanked (floxed) alleles of *Jnk1* (*Jnk1*^FL^) and *Jnk2* (*Jnk2*^FL^) were crossed to *alfp*-Cre transgenic mice to generate a liver parenchymal cell-specific knockout (LPC-KO) of both genes (6–8). Double-knockout mice (JNK1/2^LPC-KO^) were generated by intercrossing JNK1^LPC-KO^ and JNK2^LPC-KO^ single-mutant mice. Mice with combined conditional deletions of *Jnk1*/*2* and *Caspase-8* (JNK1/JNK2/Casp-8^LPC-KO^) or additional conditional deletion of *Ripk1* (*Ripk1*^FL^) or *Mlkl* (*Mlkl*^FL^) (JNK1/JNK2/RIPK1^LPC-KO^, JNK1/JNK2/RIPK1/Casp-8^LPC-KO^, and JNK1/JNK2/MLKL^LPC-KO^) were generated by intercrossing the respective lines to JNK1/2^LPC-KO^ mice (24, 46, 47). For the generation of JNK1/2^Sox9-cre/ERT2^ mice, *Jnk1*^FL^/*Jnk2*^FL^ mice were crossed with mice carrying the gene for an estrogen receptor-dependent inducible Cre recombinase under control of the Sox9 promotor (26). Cre expression was activated by a single injection of 100 µg/g tamoxifen (solved in 10% EtOH and 90% sunflower oil) at the age of 4 to 5 wk. In all experiments, littermates carrying the respective loxP-flanked alleles but lacking expression of Cre recombinase were used as WT controls. Mice were bred on a mixed C57/BL6-SV129Ola genetic background. Only sex- and age-matched animals were compared. All animals mentioned up to this point were treated in full compliance with the guidelines for animal care approved by the Federal Ministry for Nature, Environment and Consumers’ Protection of the state of North Rhine-Westphalia and work was performed in accordance to the respective national, federal, and institutional regulations.

Mice with LPC-specific *Jnk1*/*Jnk*2 deletion, designated as JNK1/2^Δhep^, were generated by intercrossing *AlbCre*^+/−^, *Jnk1*^FL^, and *Jnk2*^FL^ mice, which have been described previously (6, 7). All intercrossings and experiments involving JNK1/2^Δhep^ mice were performed at the University of Zurich. All procedures and protocols were approved by the Cantonal Veterinary Office (Zurich, Switzerland).

More detailed information on the materials and methods used in this study is provided in *SI Appendix*, *Materials and Methods*.

## Supplementary Material

Supplementary File

Supplementary File

Supplementary File

Supplementary File

Supplementary File

Supplementary File

## Data Availability

All study data are included in the article and/or supporting information.
