# Supplementary material for: JNK signaling prevents biliary cyst formation through a CASPASE-8–dependent function of RIPK1 during aging
Source: Proc Natl Acad Sci U S A. 2021 Mar 8;118(12):e2007194118. doi: 10.1073/pnas.2007194118 (PMC8000530; doi:10.1073/pnas.2007194118)
Supplement: Supplementary File [file pnas.2007194118.sapp.pdf]

PNAS
www.pnas.org

Supplementary Information for
JNK signaling prevents biliary cyst formation through a CASPASE-8-dependent function of RIPK1 during aging.

Katrin Müller, Hanna Honcharova-Biletska, Christiane Koppe, Michèle Egger, Lap Kwan Chan, Anne T. Schneider, Lena Küsgens, Friederike Böhm, Yannick Boege, Marc E. Healy, Johannes Schmitt, Sarah Comtesse, Mirco Castoldi, Christian Preisinger, Marta Szydlowska, Enrico Focaccia, Nadine T. Gaisa, Sven Loosen, Simone Jörs, Frank Tacke, Christoph Roderburg, Verena Keitel, Johannes G. Bode, Peter Boor, Roger J. Davis, Thomas Longerich, Fabian Geisler, Mathias Heikenwalder, Achim Weber, Mihael Vucur and Tom Luedde

Prof. Dr. Tom Luedde
Email: tom.luedde@med.uni-duesseldorf.de

Prof. Dr. Achim Weber
Email: achim.weber@usz.ch

This PDF file includes:

Supplementary text
Figures S1 to S4
Tables S1 to S2
Legends for Dataset 1 to Dataset 5
SI References

Other supplementary materials for this manuscript include the following:

Datasets 1 to Dataset 5

SI Material and Methods

Serum analysis. Serum ALT, GLDH, and AP activities were determined by standard procedures in the Institute of Clinical Chemistry of the RWTH University Hospital Aachen and in the Institute of Clinical Chemistry at the University Hospital Zurich.

Histology, immunohistochemistry (IHC) and in situ hybridization (ISH). Paraformaldehyde (4%) fixed and paraffin embedded liver tissues (human and mouse liver samples) were cut in sections (2 µm) and stained with H&E [hematoxylin (Dako) and eosin (Sigma-Aldrich)] or various primary and secondary antibodies.

For IHC staining, tissue sections were deparaffinized with xylene and rehydrated in graded alcohols. Antigen retrieval was performed in EDTA-buffer (10 mM Tris, 1 mM EDTA, 0.05% Tween, pH 9) or citrate buffer (180 µM citric acid, 100 mM sodium citrate) at 98°C for 30 min. The sections were then treated with 3% hydrogen peroxidase (H_2O_2) in water for 10 min to block endogenous peroxidase activity, reacted with MOM Kit (Vector Laboratories) (for pan-CK staining) and then unspecific binding sites were blocked with normal horse serum (Vector Laboratories) for 30 min. IHC staining was performed with the following primary antibodies at 4°C overnight: anti-Ki67 (Thermo Scientific, 1:2000), anti-pan-cytokeratin (pan-CK) (Thermo Scientific, 1:2000), anti-RIPK1 (Novus Bio, 1:2000), and anti-γH2A.X (Cell Signaling, 1:600). Sections were then incubated with the secondary antibodies: anti-rabbit IgG HRP and anti-mouse IgG HRP (Vector Laboratories) and bound antibodies were visualized with 3-3´-diamonobenzidine (DAB, Thermo Scientific). For double stainings a second primary antibody was used and visualized with Fast Red in Naphtol Substrate (Thermo Scientific). All sections were counterstained with hematoxylin (Dako).

Image acquisition was performed on the Slide Scanner Aperio AT2 (Leica). Automated immunohistochemical stainings, image acquisition, and quantification were performed as previously described (1). The following antibody was used: anti-cleaved caspase-3 (Cell Signaling; 1:300).

Immunohistochemistry on human liver samples was performed on liver tissues from patients with hereditary polycystic diseases (Caroli disease/syndrome n = 9, ADPKD n = 3, ARPKD n = 3, biliary microhamartoma n = 6). Tissues were provided by the Tissue Bank of the National Center for Tumor Disease (NCT) Heidelberg, Germany in accordance with the regulations of the tissue bank and the approval of the ethics committee of Heidelberg University (206/2005). Immunohistochemistry was performed. RIPK1 immunohistochemistry (dilution 1:600; Novus Biologicals, UK) was performed on 5 µm sections after antigen retrieval using citrate buffer (pH 6.1; Dako, Glostrup, Denmark).

RNA ISH for *Mcl1, Jnk1, Jnk2* on FFPE liver tissues was performed according to the manufacturer`s protocol basically as described using three commercially available probes: MAPK8/JNK1, MAPK9/JNK2 by Advanced Cell Diagnostics (ACD). Slides were evaluated by light microscopy and scanned for evaluation by digital microscopy (NDPI viewer) (2).

Proteome analysis. Cyst fluid isolates were separated by SDS-PAGE. The gels were stained with Coomassie Brilliant Blue. 8 gel slices per lane (two lanes in total) were excised and subjected to in-gel tryptic digestion (3). After digestion, the samples were desalted using homemade C18 tips and dried down. The lyophilized peptides were resuspended in 15 µl 3% formic acid (FA)/5% acetonitrile (ACN) and then subjected to mass spectrometry analysis. First, the peptides were trapped on a C18 precolumn (Acclaim PepMap100, C18, 5 µm, 100 Å, 300 µm i.d. × 5 mm, Thermo Scientific) using buffer A (0.1% FA) on a nanoLC system (RSLCnano, Thermo Scientific). Subsequently, the peptides were separated on an Easyspray C18 analytical column (Thermo Scientific) coupled with the Easyspray source (2 µm particle size, 75 µm inner diameter, 50 cm length, 40°C column oven temperature, 1.9 kV spray voltage; Thermo Scientific) 90-minute gradients. The applied gradient was as follows: 0–10 minutes, 5% buffer B (80% ACN/0.1% FA); 10–45 minutes, 5%–35% buffer B; 45–55 minutes, 35%–50% buffer B; 55–58 minutes, 50%–90% buffer B; 58–63 minutes, 90% buffer B; 63–64 minutes, 90%–5% buffer B; 64–90 minutes 5% buffer B. Mass spectrometry was performed on a Q Exactive Plus instrument (Thermo Scientific) in data-dependent mode. Full MS settings: resolution: 70,000; AGC target, 3e6; maximum injection time: 100 milliseconds; scan range: 300–1750 m/z. dd-MS2 settings: resolution: 17,500; AGC target: 2e5; maximum injection time: 110 milliseconds; fragmentation of the top 10 precursor; isolation

window: 1.8 m/z; collision energy: 27. dd settings: minimum AGC, 5e2; dynamic exclusion: 10 seconds; only 2+ to 5+ peptides were allowed.
The raw data was analyzed using MaxQuant (1.6.1.0) and the built-in Andromeda search engine (4). The spectra were searched against the mouse SwissProt database (version 06/2018) (only reviewed and canonical sequences), including the contaminants function of MaxQuant. MaxQuant default settings (including mass tolerance) were used. Specific settings included trypsin as the specific protease (2 missed cleavages); fixed modification: carbamidomethylation; variable modifications: oxidation (Met) and N-terminal protein acetylation; the false discovery rate was set to 0.01 for both peptide and protein levels; minimum peptide length, 7 amino acids.
The proteinGroups.txt results derived from the MaxQuant search were further analyzed using Perseus (5). "Potential contaminants, "reverse" and "only identified by site" hits were excluded. Further requirements for protein inclusion was a minimum of two peptides (with a minimum of 1 unique peptide). Proteins were only included in the final data set if they were identified in both lanes of cyst fluid (based on the intensity value).

16S rDNA PCR. The 16S rDNA PCR was performed using a pair of primers (Forward primer (5′-ACTCCTACGGGAGGCAGCAGT -3′) and reverse primer, (5′- ATTACCGCGGCTGCTGGC -3′)) universal to all bacterial groups (6). For each sample, 10 ng of the isolated genomic DNA were added to 1x PCR reagent mixture (1 U HotStarTaq polymerase (Qiagen), 1x CoralLoad PCR buffer (Qiagen), 200 nM dNTP, 200 nM of primers). PCR was performed using a C1000 Touch Thermal Cycler (Biorad) using the protocol as follows: 95°C for 5 min, then 25 cycles of 94°C for 1 min, 55°C for 1.5 min and 72°C for 1 min. These cycles were followed by 72°C for 10 min, and storage at 4°C. The resulting amplicon has a size of 197 bp.

Western blot and immunoblot analysis. Liver tissue was homogenized in NP-40 lysis buffer [50 mM Tris-HCl (pH 7.5), 150 mM NaCl, 0.5% NP-40 supplemented with PhosSTOP™ phosphatase inhibitor (Roche), complete™ protease inhibitor (Roche), 1 mM Pefablock (Roche) and 1 mM 1,4-Dithiothreitol (DTT, Roth)] using ceramic beads and a Bead Ruptor 12 (both Omni International) to obtain protein lysates. Cell pellets were lysed in the same buffer by repeated pipetting. To generate the soluble fraction of the lysates, the samples were centrifuged at 14000 rpm for 10 min at 4°C and the soluble supernatants retained. The supernatant was further boiled 5 min with 2x laemmli sample loading buffer (BIORAD). 60 µg of protein per sample were separated by SDS-polyacrylamide gel electrophoresis (PAGE), transferred to PVDF membrane (Merck Millipore) and analyzed by immunoblotting as previously described (Luedde et al., 2003). Membranes were probed with the following antibodies: anti-cleaved-caspase-3 (Cell Signaling), anti-JNK1 (Cell Signaling), anti-β–Actin (Cell Signaling, 1:2500), anti-JNK2 (Cell Signaling), anti-SAPK/JNK (Cell Signaling), anti-caspase-8 (Enzo Lifesciences), anti-MLKL (BiOrbyt), anti-RIPK1 (BD) and anti-GAPDH (AbD Serotec). As secondary antibodies, anti-rabbit-HRP, anti-mouse-HRP (GE Healthcare); and anti-rat-HRP (Santa Cruz) were used.

Analysis and quantification of immunohistochemical stainings. For the analysis for LPC-KO mice the quantification of liver cyst areas was done by scanning and analyzing whole tissue sections by ImageJ (Fiji). The pixels covered by the whole tissue section and the empty space (white area) were quantified. For quantification of proliferating hepatocytes, positive stains for Ki67 were counted manually and normalized to tissue area in five non-overlapping and randomly selected pictures (0,8 mm^2 each) per histological slide using ImageScope Software. Positively stained cholangiocytes (double positive for Ki67 and pan-CK) were counted manually and normalized to the total number of pan-CK positive counts in the corresponding images. ImageScope Software was used for the quantification of Ki67 and γH2A.X.

Immunofluorescence (IF) and IHC (for 3D imaging and 3D analysis). Animals were perfused transcardially with 40 ml PBS and 40 ml 4% PFA (7 ml/min). By default, the left liver lobe was processed for standard histological and IHC analysis whereas the median, right lower, and right upper liver lobes were processed for 3D IF analysis. For standard histological and IHC analysis, dissected livers were post-fixed in 4% PFA for 24 h, embedded in paraffin, and sectioned. Serial 3.5 µm-thick sections were stained with primary antibodies followed by diaminobenzidine staining

(Vector Laboratories) and counterstaining with hematoxylin. For 3D imaging, perfused livers were post-fixed in 4% PFA for 2 h at 4ºC, washed in PBS, cryoprotected with increasing concentrations of 10%, 20%, and 30% sucrose solutions prepared in PBS, and embedded in O.C.T. Tissue Tek compound. For 3D fluorescent imaging liver lobes were cut into 200 µm-thick sections using a cryostat-microtome. Free-floating tissue-sections were washed 4× 15 min with 1× PBS, permeabilized with 0.5% Triton X-100 in PBS for 1h at RT, and blocked in 0.5% Triton X-100, 10% serum in PBS for 2 h at 37°C and at 4°C overnight. Pretreated tissue slices were incubated with primary antibody dilution in 0.5% Triton X-100, 5% Serum in PBS at 4°C for 2-3 days. After thorough washing, samples were incubated with fluorescent secondary antibodies and with 4,6-diamidino-2-phenylindole (DAPI, Sigma) for nuclear co-staining diluted in 0.5% Triton X-100, 5% serum in PBS at 4°C for 2-3 days. Sections were finally washed with PBS and stored at 4°C in PBS until clearing. All incubation and washing steps were performed on a shaking device.

Tissue clearing, 3D imaging, and 3D analysis. Immunolabeled liver sections were cleared with a modified version of 3DISCO protocol (7). All steps were performed at RT on a shaking device. Samples were incubated successively in 50% (v/v), 75% (v/v), and 100% (v/v) tetrahydrofuran (THF, Sigma) for 15 min each in a glass vial. Finally, samples were immersed in dibenzyl ether (DBE, Sigma) for at least 10 min. Cleared liver sections were mounted in clearing solution (DBE refraction index: ~1.56). Immunofluorescence micrographs and z-stacks were acquired with confocal microscopy (Leica TCS SP8) equipped with Diode 405, white light laser, and HC PL APO CS2 20×/0.75 IMM oil objectives. Microscope settings were: 1024 × 1024 pixel frame size; 400 Hz scan speed; 1 µm z-step size; Pinhole 1.00 AU; sequential scan between stacks. 3D analysis was performed with IMARIS 8.3 software (Bitplane). For visualization of volume images, gamma corrections, and median filtering (3×3×3) were applied to optimize staining intensities and to remove noise or artifacts. Furthermore, 3D surface reconstructions and resulting number of connected surfaces were constructed using the “Surface” function.

Cell culture. Primary mouse cholangiocytes (Cell Biologics) were cultured on gelatin-coated tissue culture plastic in complete epithelial cell medium (Cell Biologics) containing FCS (fetal calf serum), L-glutamine, antibiotics, EGF (epidermal growth factor) and ITS (insulin-transferrin-serin). Inactivation of JNK was achieved by adding 50 µM JNK inhibitor SP600125 (Selleckchem) to the culture medium and incubating for 2h prior to further experiments. The corresponding amount of DMSO was used as control.

For stimulation of TNF signaling cells were treated with 10 ng/ml murine TNFα for 1h and then harvested by scratching in cold PBS (phosphate buffered saline). Cell pellets were used for protein isolation.

Kinase activity profiling microarray. Each cell culture stimulation experiment was performed three times independently and samples were measured as biological replicates with n = 3.

Frozen cell pellets of stimulated cells were resuspended in M-PER mammalian protein extraction reagent supplemented with PhosphoSTOP (Roche) and cOmplete Tablets (Roche), incubated on ice for 15 minutes. After centrifugation at 20.000x g for 15 minutes at 4°C the protein concentration in the supernatant is evaluated via Bradford assay. The lysate is aliquoted, snap frozen and stored at -80°C. Only unthawed aliquots are used for the kinase activity assay.

Ser/Thr Kinase (STK) activity profiling assays based on measuring peptide phosphorylation by protein kinases (PamGene International BV, The Netherlands) were performed as instructed by manufacturer. In summary, samples with 1 µg protein were applied on PamChip®4 arrays containing 144 (STK) or 196 (PTK) peptides immobilized on a porous aluminum oxide membrane. The peptide sequences (13 amino acids long) harbor phosphorylation sites and are correlated with one or multiple upstream kinases. Fluorescently labelled anti-phospho antibodies are used to detect phosphorylation activity of kinases present in the sample (8, 9). Instrument operation and imaging are controlled by the EVOLVE 2.0 software and quantified using BioNavigator 6.3 (BN6; PamGene International BV, The Netherlands). Signal intensities at multiple exposure times were integrated by linear regression (S100), Log2-transformed, and normalized using a Combat correction model for batch correction where the scaling parameters (mean / sd) are estimated using an empirical Bayes approach, which effectively reduces the noise associated with applying the

correction (10, 11). The normalized values were used to perform statistics comparing 2 groups using unpaired t-tests or the upstream kinase analysis (UKA) tool (BN6; PamGene international BV).

The phylogenetic kinome tree, useful to group the kinases into sequence families, is plotted using the online portal CORAL: http://phanstiel-lab.med.unc.edu/CORAL/ (12). The upstream kinase analysis functional scoring tool (PamGene International) rank-orders the top kinases differential between the two groups, the ranking factor being the final (median) kinase score (represented by node size). This score is based on a combined sensitivity score (difference between treatment and control groups, represented as node color) and specificity score for a set of peptides to kinase relationship that are derived from existing databases. An arbitrary threshold of a final score of 1.2 was applied, based on the specificity scores.

Significant peptides (t-tests, p-value < 0.05)) or kinases (UKA, final scores > 1.2) were imported to the MetaCore pathway analysis tool (Clarivate Analytics) where an enrichment analysis was performed for pathways and networks. It consists of matching the kinases or substrates in the kinome arrays data with functional ontologies in MetaCore. The probability of a random intersection between a set of IDs in the target list with ontology entities is estimated in p value of hypergeometric intersection. The lower p value means higher relevance of the entity to the dataset, which shows in higher rating for the entity. Direct interaction network algorithms were used to build interconnected networks within each comparison, and the "Add interactions" feature was used to add the interaction between RIPK1 and the data present in the MetaCore™ database after it was built.

Human samples. Analysis of anonymized human liver tissues was approved by the local ethics committee ("Kantonale Ethikkommission Zurich", application number StV26/2005 and KEK-Zh-Nr. 2013-0382).

Statistics. Mouse data were analyzed using PRISM software (GraphPad Prism; GraphPad Software) and are expressed as mean. Gaussian distribution was tested with Kolmogorov-Smirnov test. Differences between two groups were assessed by an unpaired two-sample t test or Mann-Whitney test and multiple comparisons between more than two groups have been conducted by ANOVA with Bonferroni test or Kruskal-Wallis test for post hoc analysis.

Data availability. All raw data is included in the corresponding tables and datasets.

SI Figures

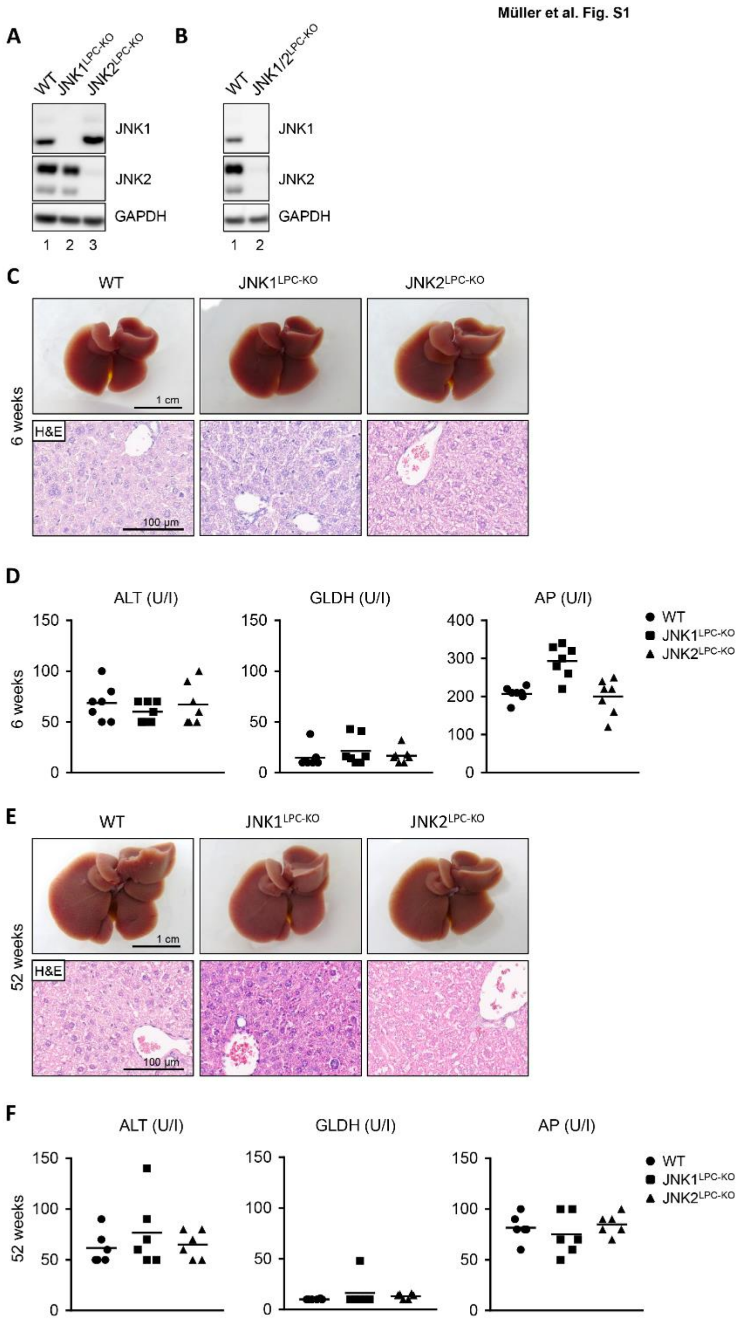

Fig. S1. Analysis of the function of JNK1 and JNK2 in the liver. (A) Western blot analysis on whole liver extracts of 6 weeks old WT, JNK1$^{LPC\text{-}KO}$, and JNK2$^{LPC\text{-}KO}$ animals. (B) Western blot analysis on whole liver extracts of 6 weeks old WT and JNK1/2$^{LPC\text{-}KO}$ animals. (C) Representative macroscopic liver pictures (top) and HE stainings of liver sections (bottom) of 6 weeks old WT, JNK1$^{LPC\text{-}KO}$, and JNK2$^{LPC\text{-}KO}$ animals. (D) Serum analysis of ALT, GLDH, and AP in 6 weeks old WT, JNK1$^{LPC\text{-}KO}$ and JNK2$^{LPC\text{-}KO}$ mice. n = 7. (E) Representative macroscopic liver pictures (top) and HE stainings of liver sections (bottom) of 52 weeks old WT, JNK1$^{LPC\text{-}KO}$, and JNK2$^{LPC\text{-}KO}$ animals. (F) Serum analysis of ALT, GLDH, and AP in 52 weeks old WT, JNK1$^{LPC\text{-}KO}$ and JNK2$^{LPC\text{-}KO}$ mice. n = 6.

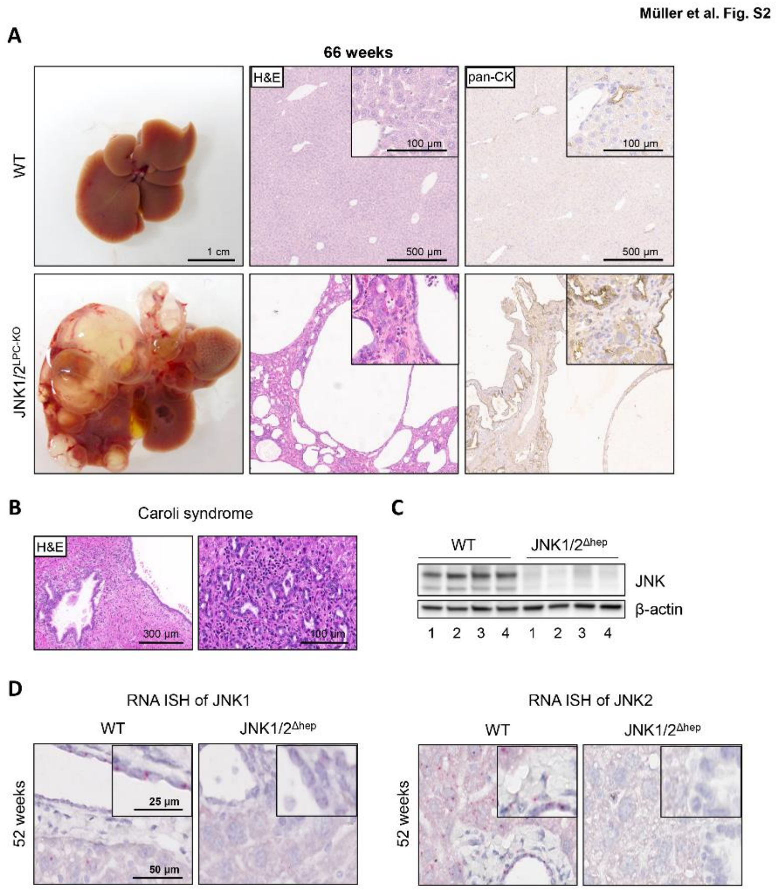

Genotype	Positive hepatocytes (%)	Positive biliary cells (%)
WT	53	32
JNK1/2$^{\Delta hep}$	<1	<1

Genotype	Positive hepatocytes (%)	Positive biliary cells (%)
WT	48	44
JNK1/2$^{\Delta hep}$	0.7	3.3

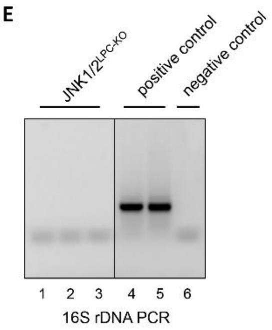

Fig. S2. Aged JNK1/2$^{LPC\text{-}KO}$ and JNK1/2$^{\Delta hep}$ mice develop severe liver cysts resembling the clinical appearance of human Caroli syndrome. (A) Representative macroscopic liver pictures (left), HE stainings (middle) and pan-CK stainings (right) of 66 weeks old WT and JNK1/2$^{LPC\text{-}KO}$ mice. (B) Representative pictures of HE sections of human liver with diseases causing cystic tissue alterations. (C) Western blot analysis on whole liver extracts of 52 weeks old WT and JNK1/2$^{\Delta hep}$ animals. (D) Representative pictures and quantification of RNA *in-situ*-hybridisation for JNK1 and JNK2 in 52 weeks old WT and JNK1/2$^{\Delta hep}$ mice. (E) Result of PCR product of bacterial 16S-DNA in 2% agarose gel electrophoresis after RNA isolation and cDNA synthesis from cyst tissue from 52 weeks and 66 weeks old WT and JNK1/2$^{LPC\text{-}KO}$ mice.

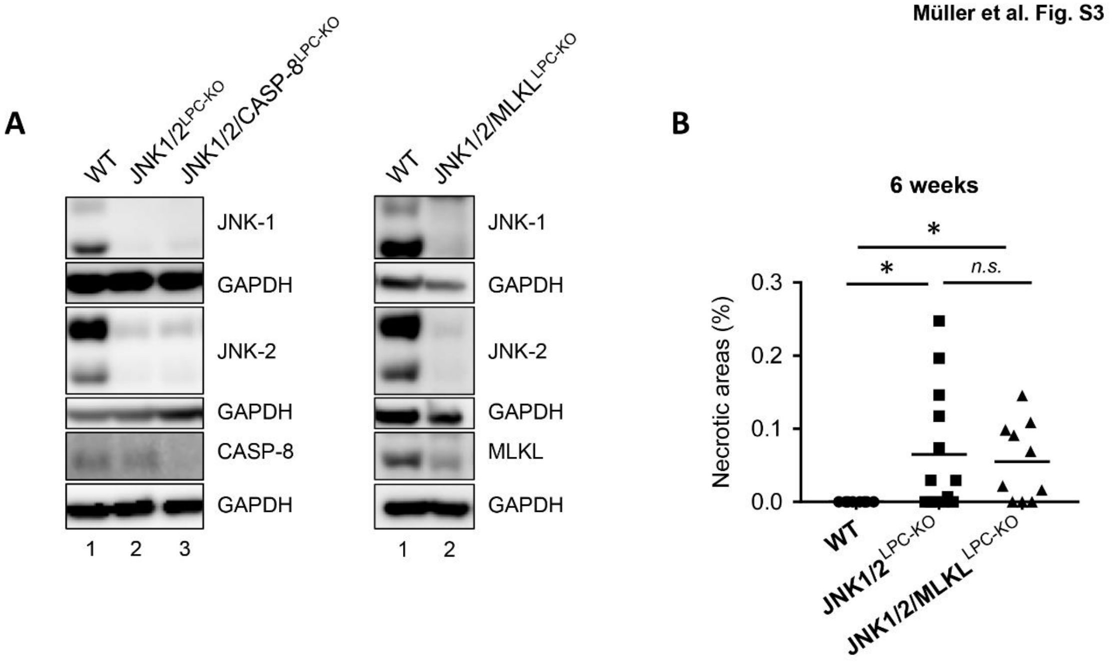

Fig. S3. Knockout confirmation for JNK1/2/Casp-8$^{LPC\text{-}KO}$ and JNK1/2/MLKL$^{LPC\text{-}KO}$ animals and persisting necrotic areas in JNK1/2/MLKL$^{LPC\text{-}KO}$ animals. (A) Western blot analysis on whole liver extracts of 6 weeks old WT, JNK1/2$^{LPC\text{-}KO}$, JNK1/2/Casp-8$^{LPC\text{-}KO}$, and JNK1/2/MLKL$^{LPC\text{-}KO}$ animals. (B) Quantification of necrotic areas in liver sections of 6 weeks old WT, JNK1/2$^{LPC\text{-}KO}$, and JNK1/2/MLKL$^{LPC\text{-}KO}$ mice. *$p < 0.05$. n = 8 – 13.

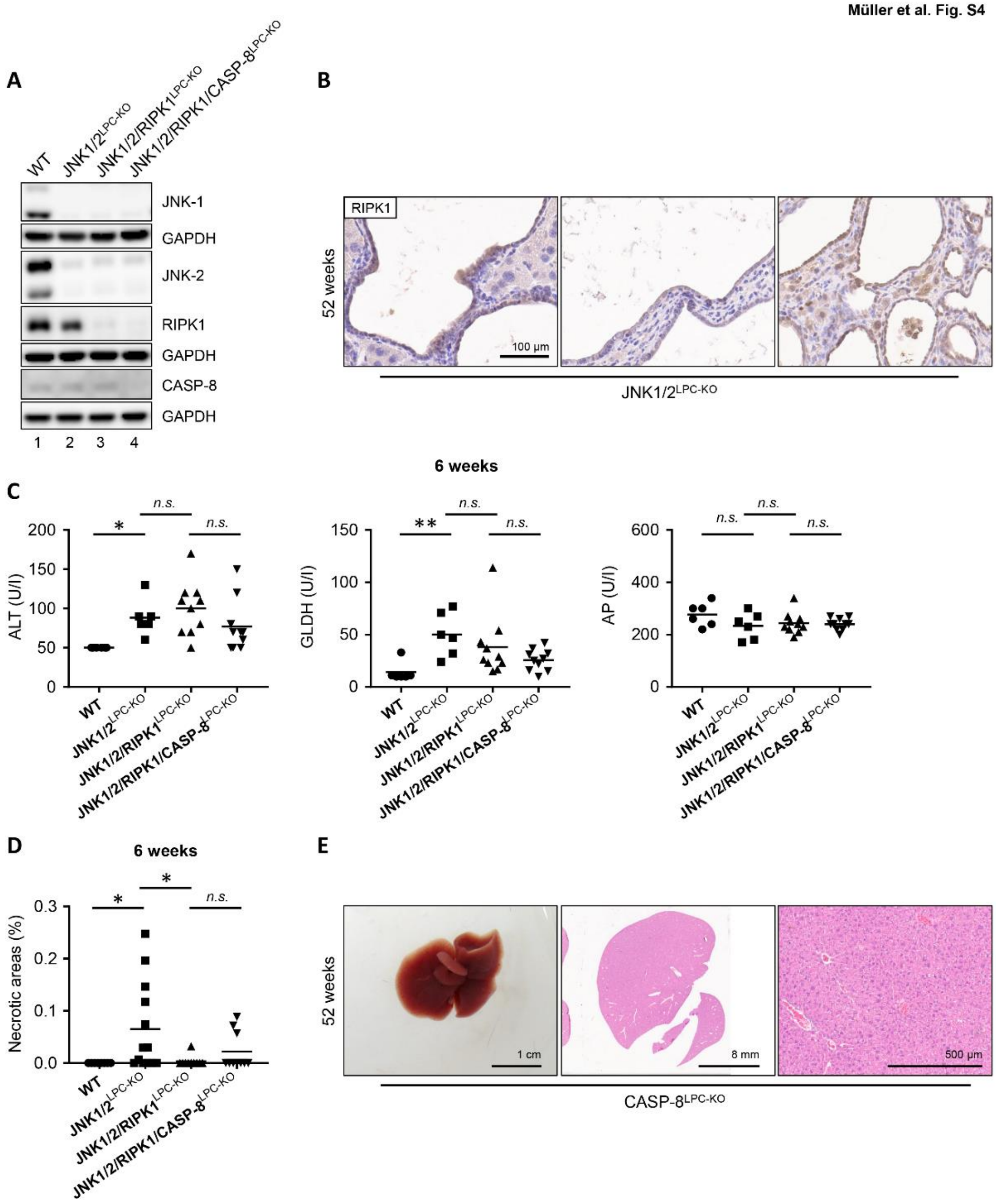

Fig. S4. Knockout confirmation and liver damage evaluation of WT, JNK1/2^LPC-KO^, JNK1/2/RIPK1^LPC-KO^, and JNK1/2/RIPK1/Casp-8^LPC-KO^ mice. (A) Western blot analysis on whole liver extracts of 6 weeks old WT, JNK1/2$^{LPC\text{-}KO}$, JNK1/2/RIPK1$^{LPC\text{-}KO}$, and JNK1/2/RIPK1/Casp-8$^{LPC\text{-}KO}$ animals. (B) Representative pictures of immunohistochemical staining of RIPK1 (brown) in liver sections of 52 weeks old JNK1/2$^{LPC\text{-}KO}$ mice. JNK1/2/RIPK1$^{LPC\text{-}KO}$ mice were used as negative control. n = 9. (C) Serum analysis of ALT, GLDH, and AP in 6 weeks old WT, JNK1/2$^{LPC\text{-}KO}$, JNK1/2/RIPK1$^{LPC\text{-}KO}$, and JNK1/2/RIPK1/Casp-8$^{LPC\text{-}KO}$ mice. n = 6 - 10. (D) Quantification of necrotic areas in liver sections of 6 weeks old WT, JNK1/2$^{LPC\text{-}KO}$, JNK1/2/RIPK1$^{LPC\text{-}KO}$, and JNK1/2/RIPK1/Casp-8$^{LPC\text{-}KO}$ mice. n = 8 - 13. WT and JNK1/2$^{LPC\text{-}KO}$ data is identical to Fig. S3*B*. (E) Representative pictures of whole liver (left), HE stained cross section of one whole liver lobe (middle), and magnified HE staining of liver cross section (right) of 52 weeks old Casp-8$^{LPC\text{-}KO}$ mice. *p < 0,05; **p < 0,005; n.s. = not significant.

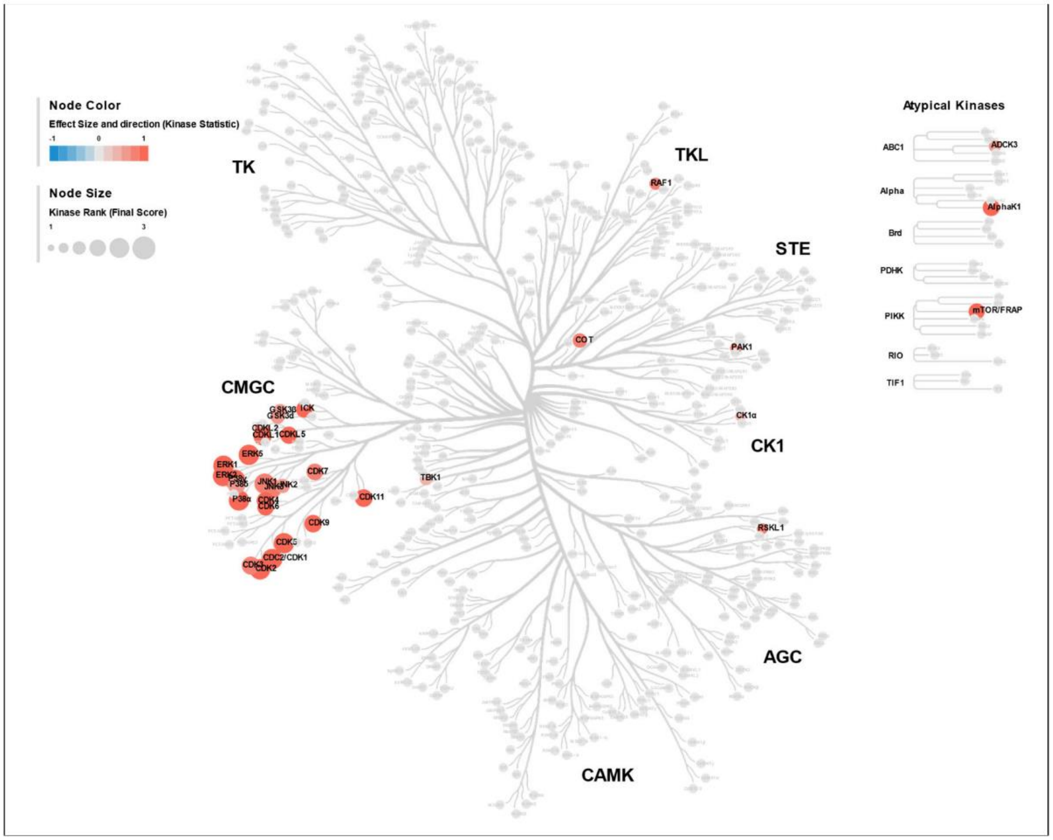

Fig. S5. Kinase activity profile upon TNF stimulation of primary mouse cholangiocytes pre-treated with solvent (DMSO). Phylogenetic kinase mapping illustrates the distribution of kinases analysed in a serine/threonine kinase activity profiling array and their regulation. Ser/Thr kinases activity, that differed upon TNF stimulation, are predominantly clustered in the CMGC-kinase family in cholangiocytes pre-treated with solvent (DMSO). The coloring scale indicating the changes in kinase activity ranges from −1 (strong decrease of kinase activity upon TNF stimulation, blue) over 0 (no change of kinase activity upon TNF stimulation, grey) to +1 (strong increase of kinase activity upon TNF stimulation, red). The node size was based on final (median) kinase score (0 to 3; 3 being largest). Serine/threonine kinase activity profiling was performed with protein lysates of cells (pre-treated with solvent (DMSO) for 2h) with or without TNF (1h) (biological replicates; n = 3).
CMGC kinase group: cyclin-dependent kinase (CDK) family, mitogen-activated protein kinase (MAPK) family, glycogen synthase kinase (GSK) family and CDC-like kinase (CLK) family.

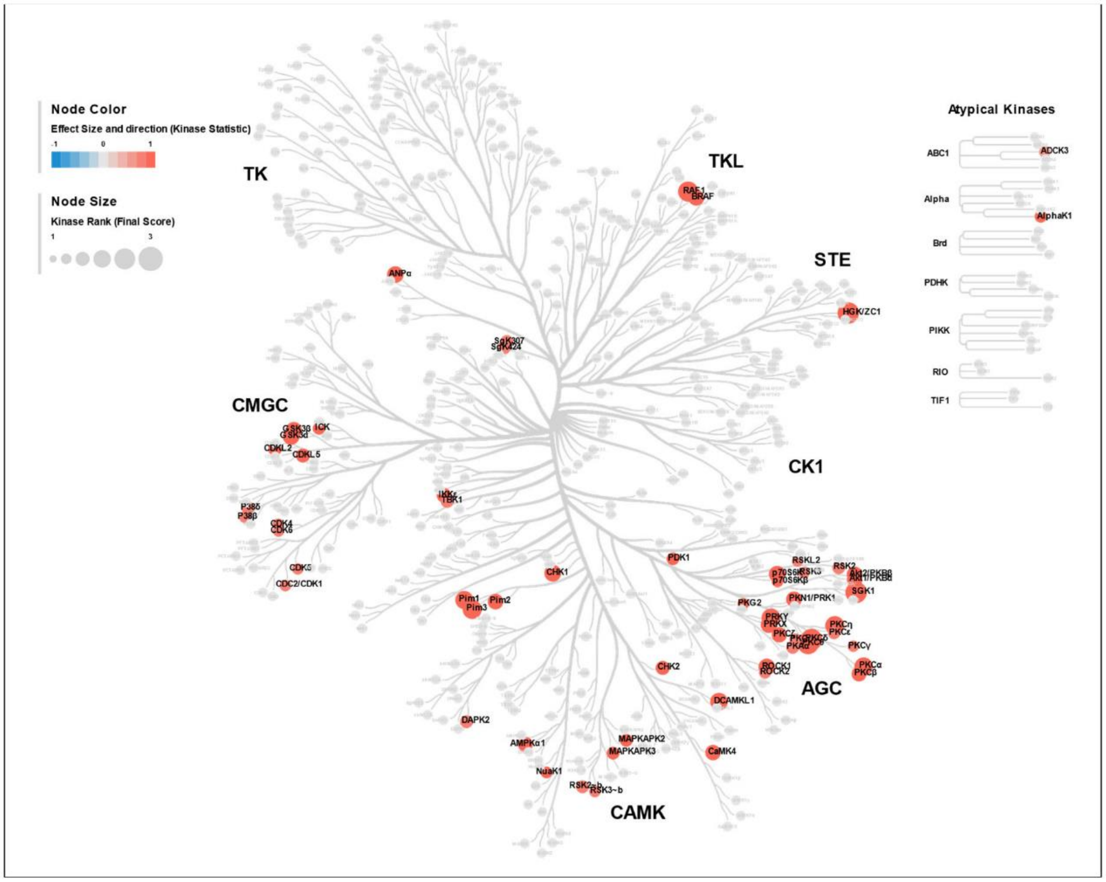

Fig. S6. Kinase activity profile upon TNF stimulation of primary mouse cholangiocytes pre-treated with JNK inhibitor (SP600125). Phylogenetic kinase mapping illustrates the distribution of kinases analysed in a serine/threonine kinase activity profiling array and their regulation. Ser/Thr kinase activity, that differed upon TNF stimulation, are predominantly clustered in the AGC-kinase family, CAMK-kinase family and CMGC-kinase family in cholangiocytes pre-treated with JNK inhibitor (SP600125). The coloring scale indicating the changes in kinase activity ranges from −1 (strong decrease of kinase activity upon TNF stimulation, blue) over 0 (no change of kinase activity upon TNF stimulation, grey) to +1 (strong increase of kinase activity upon TNF stimulation, red). The node size was based on final (median) kinase score (0 to 3; 3 being largest). Serine/threonine kinase activity profiling was performed with protein lysates of cells (pre-treated with JNK inhibitor SP600125 for 2h) with or without TNF (1h) (biological replicates; n = 3).
AGC kinase group: cAMP-dependent protein kinase (PKA), the cGMP-dependent protein kinase (PKG), and the protein kinase C (PKC) families; CAMK group: Ca2+/calmodulin-dependent protein kinase class of enzymes; CMGC kinase group: cyclin-dependent kinase (CDK) family, mitogen-activated protein kinase (MAPK) family, glycogen synthase kinase (GSK) family and CDC-like kinase (CLK) family.

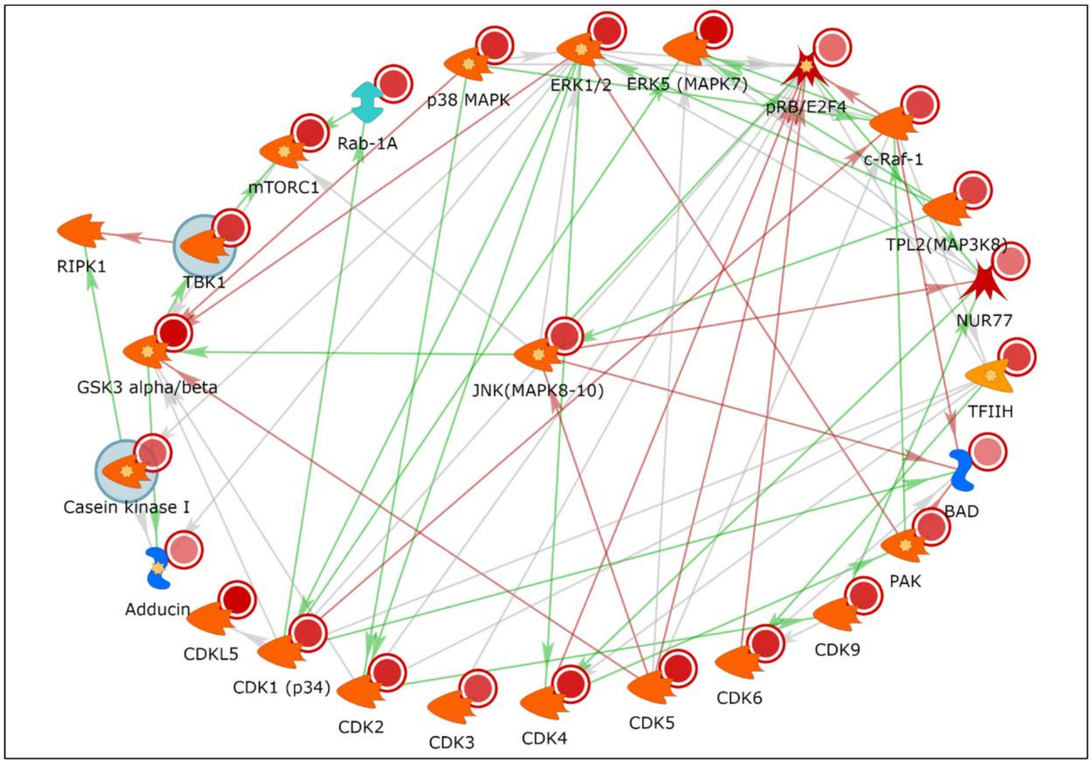

Fig. S7. Direct interaction network analysis of substrates and kinases being activated upon TNF stimulation in primary mouse cholangiocytes pre-treated with solvent (DMSO). Significant peptides (t-tests, p-value < 0.05) or kinases (UKA, final scores > 1.2) were imported to the MetaCore pathway analysis tool (Clarivate Analytics) where an enrichment analysis was performed for pathways and networks. It consists of matching the kinases or substrates in the kinome arrays data with functional ontologies in MetaCore. Direct interaction network algorithms were used to build interconnected networks within each comparison, and the "Add interactions" feature was used to add the interaction between RIPK1 and the data present in the MetaCore™ database after it was built. Node represent kinase activity increase upon TNF stimulation (TNF-stimulated versus unstimulated cells). The intensity of red color reflects the increase in kinase activity. Green arrows indicate positive interaction, red arrows indicate negative interactions and gray arrows indicate unspecified interactions. See MetaCore website for detailed legend at https://portal.genego.com/legends/MetaCoreQuickReferenceGuide.pdf .

Müller et al. Fig. S8

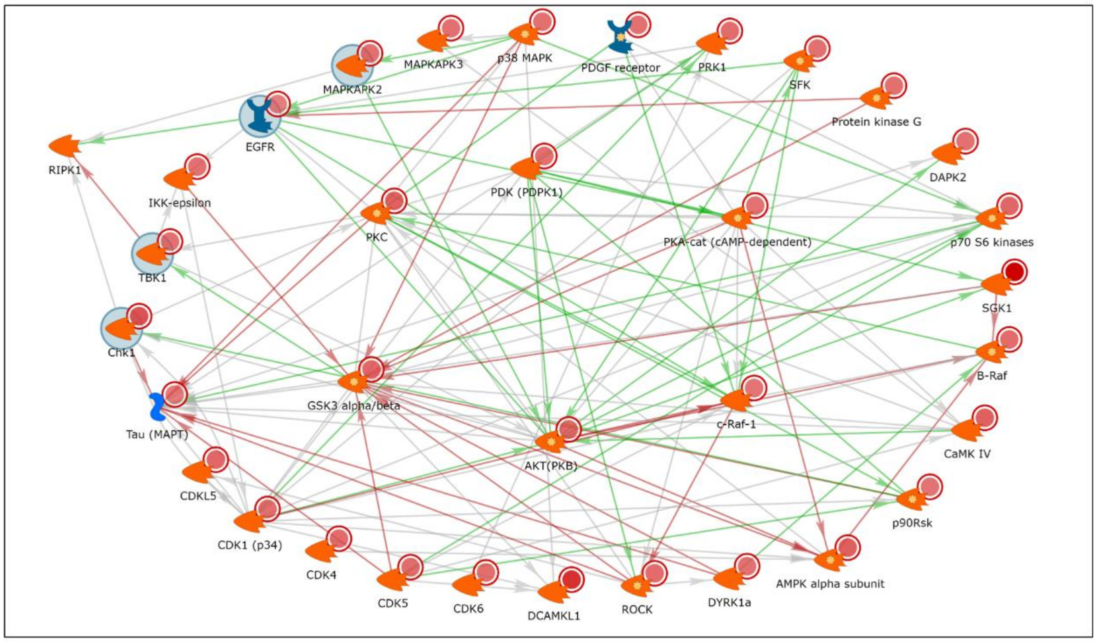

Fig. S8. Direct interaction network analysis of serine/threonine kinases being activated upon TNF stimulation in primary mouse cholangiocytes pre-treated with JNK inhibitor (SP600125). Significant peptides (t-tests, p-value < 0.05) or kinases (UKA, final scores > 1.2) were imported to the MetaCore pathway analysis tool (Clarivate Analytics) where an enrichment analysis was performed for pathways and networks. It consists of matching the kinases or substrates in the kinome arrays data with functional ontologies in MetaCore. Direct interaction network algorithms were used to build interconnected networks within each comparison, and the “Add interactions” feature was used to add the interaction between RIPK1 and the data present in the MetaCore™ database after it was built. Node represent kinase activity increase upon TNF stimulation (TNF-stimulated versus unstimulated cells). The intensity of red color reflects the increase in kinase activity. Green arrows indicate positive interaction, red arrows indicate negative interactions and gray arrows indicate unspecified interactions. See MetaCore website for detailed legend at https://portal.genego.com/legends/MetaCoreQuickReferenceGuide.pdf .

Table S1. Proteome analysis of the cyst fluid of JNK1/2^{LPC-KO} mice. Mass spectrometry analysis of the proteome of cyst fluid from two JNK1/2^{LPC-KO} mice.

Protein	Majority protein IDs	Gene name	Intensity Ex1	Intensity Ex2	Peptides	Razor + unique peptides	Unique peptides	Sequence coverage [%]	Mol. weight [kDa]	Score	Intensity	MS/MS count
Serum albumin	P07724	Alb	2,6966E+11	2,2049E+11	64	64	59	79,4	68,692	323,31	4,9016E+11	3091
Clusterin; Clusterin beta chain; Clusterin alpha chain	Q06890	Clu	95851100000	1,2868E+11	29	29	29	47,5	51,655	323,31	2,2453E+11	1542
Serotransferrin	Q921I1	Tf	1,0283E+11	1,1996E+11	58	58	55	68	76,723	323,31	2,2279E+11	1660
Complement C3	P01027	C3	75945100000	71570900000	102	102	102	60	186,48	323,31	1,4752E+11	2309
Alpha-1-antitrypsin 1-4	Q00897	Serpina1d	37763000000	29581000000	19	19	8	47,2	45,998	323,31	6,7345E+10	454
Apolipoprotein E	P08226	Apoe	21081000000	19530000000	19	19	19	47,9	35,866	323,31	4,0611E+10	386
Hemoglobin subunit alpha	P01942	Hba	678160000	30805000000	7	7	5	50,7	15,085	115,35	3,1483E+10	238
Vitamin D-binding protein	P21614	Gc	14724000000	15518000000	30	30	30	74,4	53,6	323,31	3,0241E+10	422
Ceruloplasmin	Q61147	Cp	13441000000	13827000000	48	48	44	44,8	121,15	323,31	2,7268E+10	814
Alpha-1-antitrypsin 1-3; Alpha-1-antitrypsin 1-1	Q00896;P07758	Serpina1c;Serpina1a	14458000000	12515000000	18	9	8	39,1	45,823	289,59	2,6973E+10	225
Hemoglobin subunit beta-1	P02088	Hbb-b1	661050000	23853000000	9	9	3	60,5	15,84	290,14	2,4514E+10	296
Serine protease inhibitor A3K	P07759	Serpina3k	11612000000	11066000000	18	18	11	42,3	46,879	323,31	2,2678E+10	437
Transthyretin	P07309	Ttr	11682000000	10500000000	8	8	8	65,3	15,776	238,45	2,2182E+10	136
Ig alpha chain C region	P01878		12724000000	6922000000	12	12	12	51,7	36,875	323,31	1,9646E+10	142
Protein AMBP; Alpha-1-microglobulin	Q07456	Ambp	8314510000	9276810000	15	15	15	39,5	39,029	323,31	1,7591E+10	312
Actin, cytoplasmic 2; Actin, cytoplasmic 2, N-terminally processed	P63260	Actg1	7578890000	7124700000	27	27	1	85,1	41,792	323,31	1,4704E+10	491
Alpha-1-antitrypsin 1-2	P22599	Serpina1b	7518100000	6721590000	17	7	7	36,8	45,974	238,49	1,424E+10	111
Osteopontin	P10923	Spp1	7136690000	6933300000	11	11	11	52,7	32,459	323,31	1,407E+10	177
Carboxylesterase 1C	P23953	Ces1c	6186000000	6137110000	17	17	15	33,8	61,055	233,51	1,2323E+10	198
Polymeric immunoglobulin receptor;Secretory component	O70570	Pigr	8064510000	3656800000	21	21	21	31,8	84,998	323,31	1,1721E+10	211
Alpha-2-HS-glycoprotein	P29699	Ahsg	4953300000	5974400000	10	10	10	38	37,325	323,31	1,0928E+10	117
Alpha-1-antitrypsin 1-5	Q00898	Serpina1e	5981700000	4605000000	16	9	9	39	45,891	286,75	1,0587E+10	112
Fibronectin; Anastellin	P11276	Fn1	4690300000	4821900000	62	62	62	31,4	272,53	323,31	9512300000	495
Lactadherin	P21956	Mfge8	5868500000	2680000000	25	25	25	52,9	51,24	323,31	8548500000	330
Apolipoprotein A-I; Proapolipoprotein A-I; Truncated apolipoprotein A-I	Q00623	Apoa1	3349000000	4667300000	19	19	19	51,5	30,615	183,31	8016200000	186
Apolipoprotein A-IV	P06728	Apoa4	3446900000	3360600000	24	24	24	70,4	45,029	323,31	6807500000	163
Hemopexin	Q91X72	Hpx	3975000000	2831600000	21	21	21	46,1	51,317	323,31	6806600000	289
Kininogen-1; Kininogen-1 heavy chain;Bradykinin;Kininogen-1 light chain	O08677	Kng1	3115200000	3502800000	19	19	19	33,1	73,101	323,31	6617900000	245
Lactotransferrin	P08071	Ltf	4597400000	1668000000	39	39	39	58	77,837	323,31	6265400000	215
Lysozyme C-2	P08905	Lyz2	1940000000	3764200000	8	8	3	39,2	16,689	99,421	5704300000	55

Column header	Explanation
Protein names	Protein names
Majority protein IDs	UniProt IDs of the proteins correponding to the corresponding entries
Gene names	Gene names
Intensity Ex1	Total intensity of the protein in experiment/ lane 1
Intensity Ex2	Total intensity of the protein in experiment/ lane 2
Peptides	Number of all peptides identified for the corresponding entry
Razor + unique peptides	Number of razor &unique peptides identified for the corresponding entry
Unique peptides	Number of unique peptides identified for the corresponding entry
Sequence coverage [%]	Sequence coverage [%]
Mol. weight [kDa]	Mol. weight [kDa]
Score	Andromeda score
Intensity	Sum of columns 1 and 2
MS/MS count	Total number of MS/MS counts of all peptides belonging to this entry

Table S2. Evaluation of histological RIPK1 staining on liver tissue of human PLD patients. ADPLD: autosomal dominant polycystic liver disease; ARPLD: autosomal recessive polycystic liver disease (congenital hepatic fibrosis); vMC: von Meyenburg complex; staining: - = negative, + = weak, ++ = moderate

Müller et al. Tab. S2

Entity	RIPK1 expression
ADPLD #1	-
ADPLD #2	-
ADPLD #3	+ to -
ARPLD #1	-
ARPLD #2	+
ARPLD #3	-
Caroli #1	++ to -
Caroli #2	+ to -
Caroli #3	++ to -
Caroli #4	+ to -
Caroli #5	++
Caroli #6	++
Caroli #7	+ to -
Caroli #8	++ to -
Caroli #9	+ to -
vMC #1	+ to -
vMC #2	+ to -
vMC #3	+ to -
vMC #4	+ to -
vMC #5	+ to -
vMC #6	-

Dataset S1 (separate file). Cyst fluid proteome analysis data

Dataset S2 (separate file). Kinase activity profiling microarray_Combat normalized data.

Dataset S3 (separate file). Kinase activity profiling microarray_Upstream Kinase data_Coral_TNFvs0.

Dataset S4 (separate file). Kinase activity profiling microarray_TNFvs0 DMSO Combined Metacore.

Dataset S5 (separate file). Kinase activity profiling microarray_TNFvs0 JNK inhibitor Combined Metacore.

SI References

1. C. Koppe *et al.*, IkappaB kinasealpha/beta control biliary homeostasis and hepatocarcinogenesis in mice by phosphorylating the cell-death mediator receptor-interacting protein kinase 1. *Hepatology* **64**, 1217-1231 (2016).
2. D. Lenggenhager *et al.*, Visualization of hepatitis E virus RNA and proteins in the human liver. *Journal of hepatology* **67**, 471-479 (2017).
3. A. von Kriegsheim, C. Preisinger, W. Kolch, Mapping of signaling pathways by functional interaction proteomics. *Methods Mol Biol* **484**, 177-192 (2008).
4. S. Tyanova, T. Temu, J. Cox, The MaxQuant computational platform for mass spectrometry-based shotgun proteomics. *Nat Protoc* **11**, 2301-2319 (2016).
5. S. Tyanova *et al.*, The Perseus computational platform for comprehensive analysis of (prote)omics data. *Nat Methods* **13**, 731-740 (2016).
6. D. C. Propheter, A. L. Chara, T. A. Harris, K. A. Ruhn, L. V. Hooper, Resistin-like molecule beta is a bactericidal protein that promotes spatial segregation of the microbiota and the colonic epithelium. *Proceedings of the National Academy of Sciences of the United States of America* **114**, 11027-11033 (2017).
7. A. Erturk *et al.*, Three-dimensional imaging of solvent-cleared organs using 3DISCO. *Nat Protoc* **7**, 1983-1995 (2012).
8. R. Hilhorst *et al.*, Peptide microarrays for profiling of serine/threonine kinase activity of recombinant kinases and lysates of cells and tissue samples. *Methods Mol Biol* **977**, 259-271 (2013).
9. C. S. Chirumamilla *et al.*, Profiling Activity of Cellular Kinases in Migrating T-Cells. *Methods Mol Biol* **1930**, 99-113 (2019).
10. C. Chen *et al.*, Removing batch effects in analysis of expression microarray data: an evaluation of six batch adjustment methods. *PloS one* **6**, e17238 (2011).
11. W. E. Johnson, C. Li, A. Rabinovic, Adjusting batch effects in microarray expression data using empirical Bayes methods. *Biostatistics* **8**, 118-127 (2007).
12. K. S. Metz *et al.*, Coral: Clear and Customizable Visualization of Human Kinome Data. *Cell Syst* **7**, 347-+ (2018).
